# Temporal and spatial distribution trends of polio vaccine coverage in less than one-year old children in Brazil, 2011–2021

**DOI:** 10.1186/s12889-023-16192-8

**Published:** 2023-07-14

**Authors:** Tércia Moreira Ribeiro da Silva, Ana Carolina Micheletti Gomide Nogueira de Sá, Elton Junio Sady Prates, Raphael de Freitas Saldanha, Thales Philipe Rodrigues da Silva, Antônia Maria da Silva Teixeira, Mark Anthony Beinner, Suelen Rosa de Oliveira, Antonio Tolentino Nogueira de Sá, Fernanda Penido Matozinhos, Ed Wilson Rodrigues Vieira

**Affiliations:** 1 Department of Maternal and Child Nursing and Public Health, School of Nursing, Universidade Federal de Minas Gerais (UFMG), Belo Horizonte, 30190 000 Minas Gerais Brazil; 2 Oswaldo Cruz Foundation, Rio de Janeiro, Brazil; 3 Ministry of Health, Rio de Janeiro, Brazil; 4 Hospital das Clínicas da Universidade Federal de Minas Gerais, Belo Horizonte, Minas Gerais Brazil

**Keywords:** Poliovirus Vaccines, Ecological studies, Spatial analysis, Immunization programs, Immunization schedule, Brazil

## Abstract

Low polio vaccine coverage can result in the spread of Poliovirus to areas free from viral circulation. This study analyzed the temporal trends and spatial distribution of polio vaccine coverage in one year-old children in Brazil, between 2011 and 2021. Methods: This was an ecological, time-series study (2011 to 2021) with annual vaccine coverages against poliomyelitis, extracted from the Information System of the National Immunization Program from the 26 States and the Distrito Federal (DF). The percentage reductions in vaccination coverage in Brazil and in the Regions were calculated. Prais-Winsten regression models were used to analyze time series for the Regions and States, and spatial analysis identified the distribution of clusters (high-high; low-low; high-low and low-high) of vaccination coverages across Brazilian municipalities, using a 5% significance level. Results: From 2011 to 2021, the coverage of polio vaccines decreased by 29,9%. There was a progressive increase observed in clusters resulting in low vaccination coverages (140 low-low Brazilian municipalities in 2011 vs. 403 in 2021), mostly reported in the North and Northeast regions of the country. There was a downward trend in vaccination coverages in 24 of the 26 States and DF (p ≤ 0.05). Conclusions: The reduction in polio vaccine coverage, as observed in the North and Northeast regions of Brazil, may favor the spread of Poliovirus. Therefore, vaccination strategies should be prioritized for children residing in areas with sharp and recurrent declines in vaccination coverages, including travelers, migrants, and refugees.

**Supplementary Information:**

The online version contains supplementary material available at 10.1186/s12889-023-16192-8.

## Highlights

Clusters with low coverage of the poliovirus vaccine were concentrated in the North and Northeast regions of Brazil;

Poliomyelitis vaccination strategies in Brazil should be directed, in particular, to areas that present a marked and continuous decrease in vaccine coverage;

Travelers, migrants and refugees should also be included in polio vaccination strategies in Brazil.

**Supplementary Information:**

The online version contains supplementary material available at 10.1186/s12889-023-16192-8.

## Background

The Poliovirus, an Enterovirus of the Picornaviridae family, was responsible for thousands of cases of poliomyelitis worldwide until the 1960s [1, 2]. Also popularly known as polio or infantile paralysis, the transmission of Poliovirus occurs through direct contact of the susceptible individual with feces or secretions eliminated from the mouth of sick individuals [2–4]. As for the clinical manifestations, they can range from asymptomatic and mild forms, characterized by fever, malaise and headache, to severer forms, which includes acute flaccid paralysis and meningitis [1, 4].

Since 1988, there has been an intensification of immunization strategies against poliomyelitis, conducted mainly by the Global Polio Eradication Initiative (GPEI), with the ample distribution of two polio vaccines: the oral polio vaccine (OPV) and the inactivated polio vaccine (IPV) [2, 4]. The incorporation of these vaccines by health systems was responsible for a 99% reduction in the global rate of poliomyelitis world-wide, from 350,000 annual cases until the end of the 20th century [5] to 175 cases in 2019 [6]. In 1994, the Region of the Americas was the first to be declared polio-free, and in 2020, the African Continent was certified polio-free following massive vaccination campaigns conducted by the GPEI [1, 7].

However, despite the success of immunization strategies that interrupted the autochthonous transmission of wild poliovirus type 2 (WPV-2) and wild poliovirus type 3 (WPV-3) around the world, wild poliovirus type 1 (WPV-1) continues to circulate in Afghanistan and Pakistan [8]. In these countries, political and economic instability serves as a barrier to immunization actions against poliomyelitis and makes epidemiological surveillance of acute flaccid paralysis difficult, an essential strategy for early identification of the circulation of Poliovirus in the community [5, 6, 9].

It is noteworthy that sick travelers, migrants or refugees from these countries can spread WPV-1 to territories that are free of WPV-1 of this virus [10]. Added to this is the fact that low OPV coverage, in addition to the increased number of individuals susceptible to Poliovirus in certain areas, increases the chances of outbreaks of acute flaccid paralysis caused by circulation of vaccine-derived Poliovirus 2 (cVDPV2) [2, 11]. In 2020, 1,056 cVDPV2 cases were detected in Afghanistan, Pakistan, Chad and the Democratic Republic of Congo, representing triple the number of cVDPV2 cases from the previous year [7, 8]. Recently, travelers on international flights were responsible for the spread of cVDPV2 to the United Kingdom of Great Britain, Northern Ireland and the United States of America [10, 12].

The possibility of the dissemination of Poliovirus becomes even more worrisome when we evaluate the vaccination coverage of Latin American countries, once considered free of Poliovirus [13]. In 2019, the average coverage of OPV e IPV vaccines in children under one year of age in Latin America was 87% and only 13 countries achieved their target established by the World Health Organization (WHO) of 95%, a situation that has been exacerbated by the COVID-19 pandemic [13]. Under this scenario, the Regional Certification Commission classified as an elevated risk for poliomyelitis in Latin American countries that account for the worst immunization indicators: Bolivia, Brazil, Ecuador, Guatemala, Haiti, Paraguay, Suriname and Venezuela [13].

Brazil has one of the most complete immunization programs in the world, widely recognized for its immunization strategies that have ensured the reduction in incidence rates and deaths from diseases such as measles, poliomyelitis and whooping cough [14, 15]. It is noteworthy that for the prevention of poliomyelitis, the Brazilian National Immunization Program offers, free of charge, the OPV and IPV vaccines, and has established an annual target of 95% vaccination coverage of the eligible population, as recommended by the WHO [15]. However, the national reduction in vaccination coverage rates, in recent years, has resulted in a serious problem for herd immunity and the risk of resurgence of diseases once controlled or eradicated [16–18]. Additionally, in Brazil the coverage of vaccines recommended for childhood is not homogeneous, and areas with low vaccine coverage are commonly seen in the North and Northeast regions of the country [17, 19].

The identification of areas with low coverage of OPV and IPV vaccines is seen as a priority at spearheading direct immunization actions at reducing the risk in countries with Poliovirus circulation [7]. In this sense, spatial analysis can identify areas with a high number of children susceptible to Poliovirus. It can also provide health policy managers with ample information for drafting recommendations as to the necessary health policy priorities and strategies aimed at maintaining territories free from viral circulation [20]. Spatial analysis has many advantages compared to other methods commonly adopted for the analysis of vaccination coverage, which includes, the ability to visually represent the location of areas with the worst vaccination coverage indicators, using Geographic Information System tools [21]. It is noteworthy that through spatial analysis, it is possible to identify municipalities with low vaccination coverage that are close to other municipalities with the same behavior by forming clusters or spatial clusters with low vaccination coverage [21]. These low vaccination coverage spatial clusters represent the accumulation of susceptible individuals and, therefore, increased risk for the circulation of the infectious disease which is also preventable by vaccination in a given territory [14, 21].

In addition to identifying spatial clusters with a high number of children susceptible to poliomyelitis, this study analyzed temporal variations in annual coverage of polio vaccines in children under one year of age, in Brazil, from 2011 to 2020. The present study advanced, by identifying for the first time, temporal trends over a period of 11 years, as well as the spatial distribution of annual coverage of polio vaccines in children under one year of age in Brazil.

## Materials and methods

### Study design

This was an ecological and time series study with data obtained from the National Immunization Program Information System (SI-NIP, available at http://tabnet.datasus.gov.br/cgi/dhdat.exe?bd_pni/cpnibr.def) (Sistema de Informação do Programa Nacional de Imunizações, SI-PNI).

### Context

The SI-NIP provides coverage for all vaccines recommended by the National Immunization Program (NIP - Programa Nacional de Imunizações, PNI) in Brazil [14, 22]. It is noteworthy that for the prevention of poliomyelitis, the NIP offers free OPV and IPV vaccination, recommended for the primary vaccination schedule, which consists of three doses of IPV (2, 4 and 6 months) and two boosters with OPV (15 months and at 4 years of age) [23]. In this study, we chose to evaluate information from children under one year of age due to the importance of early vaccination for individuals and collective protection against Poliovirus.

SI-NIP provides three indicators of polio vaccine coverage in Brazil [23]:

1) Poliomyelitis vaccine in children under one year of age: corresponds to the proportion of children under one year of age, residing in a specific place and period, who have completed the primary vaccination schedule (three doses of vaccine against poliomyelitis, at 2, 4 and 6 months);

2) Coverage of the first Polio Vaccine booster (1 year of age): corresponds to the proportion of children aged 12 months, living in a specific place and period, who received their first booster with the vaccine against poliomyelitis at 12 months;

3) Coverage of the second Polio Vaccine booster (4 years): corresponds to the proportion of children, aged 4, living in a specific place and period, who received their second vaccine against poliomyelitis booster.

For all three indicators above, NIP established a 95% target coverage of the eligible population, as recommended by the WHO [15].

### Data collection

**Fig. 1:**
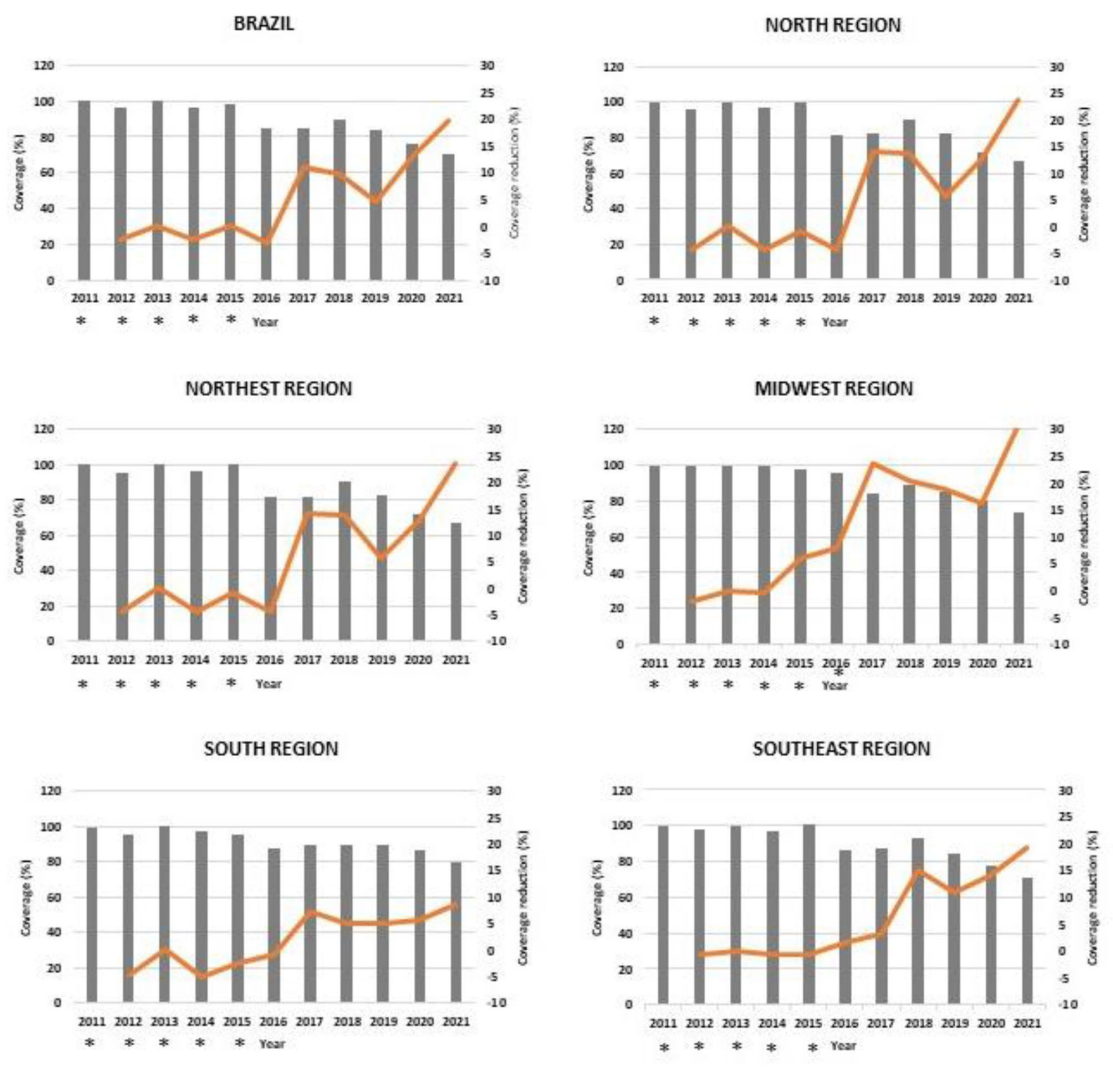
Annual evolution and percentage reduction in coverage (%) of the polio vaccine in children less than one year of age by Region in Brazil, 2011–2021. (*) Years in which the target for polio vaccine coverage were achieved

In this study, annual Polio Vaccine coverage was extracted in children under one year of age according to the following stratification: (i) polio vaccine coverage from the five Brazilian regions (North, Northeast, South, Southeast and Midwest); (ii) polio vaccine covered from the 26 Brazilian States and Distrito Federal (DF); and (iii) polio vaccine coverage in the 5,570 Brazilian municipalities between 2011 and 2021 (Fig. 1S).

The SI-NIP calculation of the annual Polio Vaccine coverage, in children under one year of age, was done using the following formula [23]:

$$\frac{Number of last doses of vaccination schedule}{Number of live births}\times 100$$

The following vaccines can be administered as the third dose as part of the primary polio vaccine schedule, and, therefore, compose the numerator of the formula above: IPV, OPV, Hexavalent (DTP, Hepatitis B, *Haemophilus influenzae* type B and IPV), or Pentavalent (DTPa, *Haemophilus influenzae* type B and IPV) [23]. Furthermore, the numbers of live births in the period were extracted from the National Information System on Live Births (Sistema de Informações sobre Nascidos Vivos, SINASC).

**Fig. 2:**
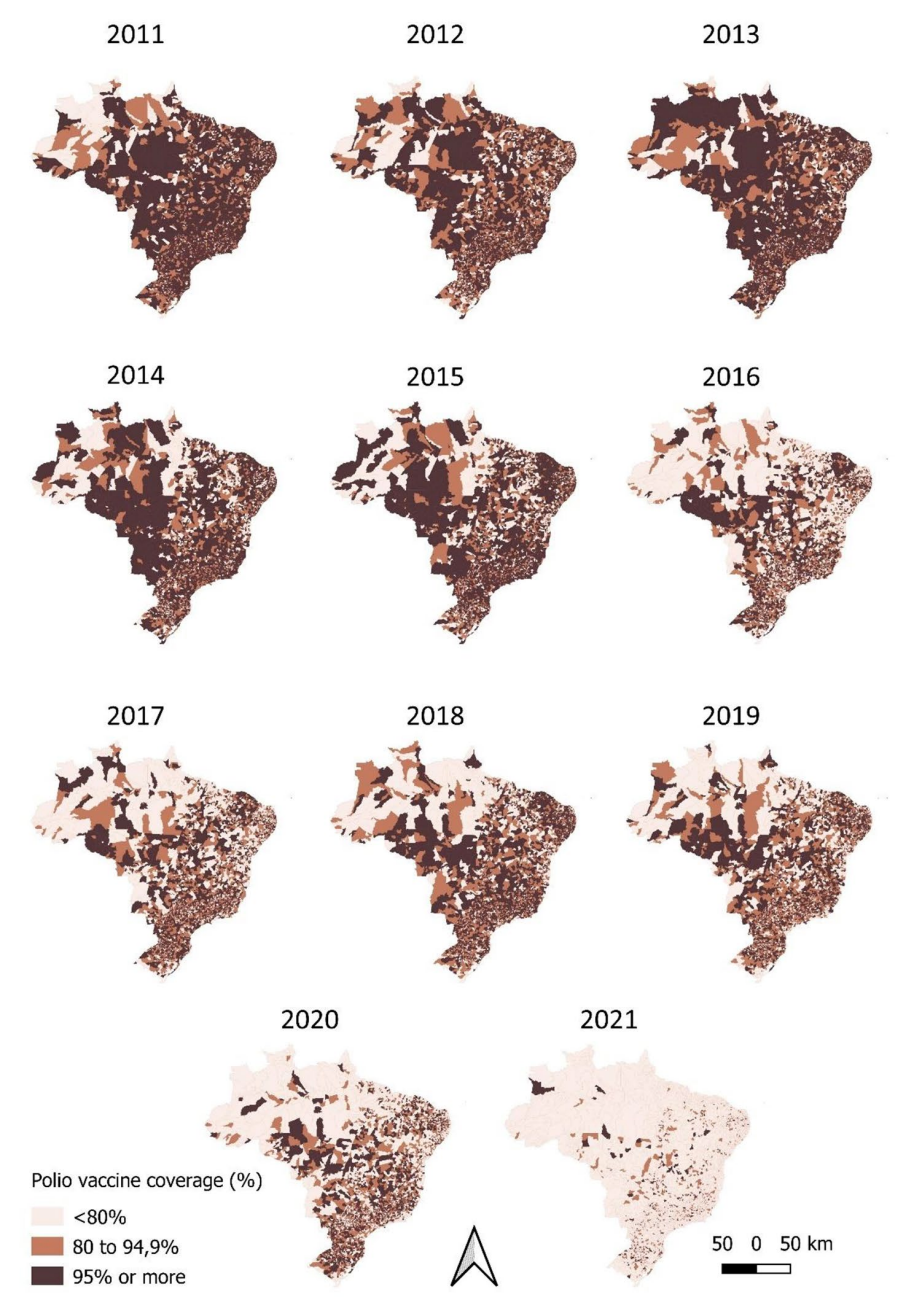
Annual evolution of coverage (%) of the polio vaccine, in children under one year old, in Brazil, according to the coverage strata of the 5570 municipalities, 2011–2021

Figure 2S presents the supplementary material formulas for calculating polio vaccine coverage (first and second boosters) (Fig. 2S).

### Data analysis and processing

For the descriptive analysis, the annual coverage of polio vaccine, in children under one year of age, according to geographic region and year (2011–2021), were evaluated.

The annual coverage of polio vaccine, in children under one year of age, was represented in bar graphs, while the annual reduction of polio vaccine coverage in children under one year of age, from 2012 onwards, with reference to the year 2011, was represented by continuous lines.

In order to analyze the spatial distribution of vaccine coverage, a spatial analysis was performed, considering the coverage of polio vaccine, in children under one year of age, as an independent variable according to each year of the evaluated period. Five regions of the country (North, Northeast, Midwest, Southeast and South), comprising 26 States and the DF, and the 5,570 Brazilian municipalities, were the dependent study variables [24].

For the descriptive spatial analysis part of this study, thematic maps were constructed from the digital notes of the 5570 Brazilian municipalities, represented by color scales and the coverage of the primary vaccination schedule against poliomyelitis. Polio vaccine coverage in children under one year of age was categorized as ideal (≥ to 95%), medium (between 80% and 94.9%) or low (≤ 80%), according to the stratification proposed from a previous study [25]. The QGIS software (version 3.18.3) was used to create the maps.

Next, spatial data analysis techniques were used, considering the digital meshes of the Brazilian municipalities. To verify whether municipalities with similar vaccination coverage formed clusters or spatial clusters, Global Moran’s I was calculated, which ranges from − 1 to + 1 with positive values indicating direct autocorrelation and negative values indicating inverse autocorrelation [26]. Spatial autocorrelation is interpreted according to the Global Moran’s I as weak (Moran’s I < 0.3), moderate (Moran’s I ≥ 0.3; < 0.7) or strong (Moran’s I ≥ 0.7) [26].

From the accessed cartographic base of Brazilian municipalities, as viewed on the IBGE website, and using two Geographic Information Programs Systems, cartograms were prepared to present the clusters with a statistical significance of p < 0.05. Using these cartograms (LISA Cluster Map type), low-low spatial clusters (dark blue) were displayed, formed by municipalities with low vaccination coverage and surrounded by municipalities with low vaccination coverage; high-high (dark red color), formed by municipalities with high vaccination coverage and surrounded by municipalities that also displayed similar behavior; high-low (municipalities with high vaccination coverage surrounded by those with low coverage), and low-high (municipalities with low coverage surrounded by municipalities with high coverage) [26].

In this study, the Moran’s I significance level of 95% was considered after 999 permutations [26], that is, the areas with statistically significant spatial correlations were those with a p-value less than or equal to 0.05 after the 999 random permutations for both Indices. The GeoDa software (version 1.20.0.8) [27] was used for this analysis.

### Trend analysis

The trends analysis of polio vaccine coverage in children under one year of age during the years 2011 to 2021 was evaluated using the Prais-Winsten autoregressive models [28]. This regression model was chosen because it allows for the correction of the autocorrelation of time series studies. The following dependent variables were adopted: (i) vaccination coverage against poliomyelitis by states and the DF; (ii) vaccination coverage by the 5 Brazilian regions, and (iii) vaccination coverage against poliomyelitis in Brazil. The independent variables were the studied years.

To avoid variance heterogeneity of the residuals gathered from the time series regression analysis, the polio vaccine coverages were transformed to logarithmic scale [28].

After pre-processing data, the annual percent change (APC) was calculated for all trend analyzes performed. The APC calculation was performed using the formula below, where the slope (*beta*) refers to b1 of the Prais-Winsten regression [28]:

$$APC=\left[-1+{10}^{b1}\right]\times 100\%$$

To calculate the 95% confidence intervals (95%CI) of the APC measurements, the following formula was used:

$$C{I}_{95\%}=\left[-1+{10}^{b1min.}\right]\times 100\%; \left[-1+{10}^{b1max.}\right]\times 100\%$$

The values of the slopes (b1) of the Prais-Winsten regression and standard errors (ep) were generated by the Statistical Software for professionals (Stata), version 16.0. In the formula, the Student’s t-test (represented by t) was also adopted, which corresponded to 10 degrees of freedom (t = 2.228 for the eleven-year period under analysis).

The trend was considered increasing when the p-value was less than 0.05 and the regression coefficient was positive. The trend was decreasing when the p-value was less than 0.05 and the regression coefficient was negative, and a stationary trend was considered when the p-value was greater than 0.05 [28].

### Ethical aspects

This study was approved by the Board of Ethics Committee of Universidade Federal de Minas Gerais, in accordance with CAAE: 51609221.4.0000.5149.

## Results

### Descriptive analysis

Between 2011 and 2021, in Brazil, polio vaccine coverage in children under one year of age decreased from 100% to 2011 to 70.1% in 2021. In the 11 years considered in this study, there was a reduction in coverage of the polio vaccine in children less than one year of age observed in the five Regions of the country (Fig. 1).

By 2015, Brazil and all regions had achieved the coverage targets for the Polio Vaccine in children under one year of age. However, in 2016, only the Midwest Region achieved the polio vaccine coverage target and, as of 2016, no region reached the coverage target for this vaccine (Fig. 1).

**Fig. 3:**
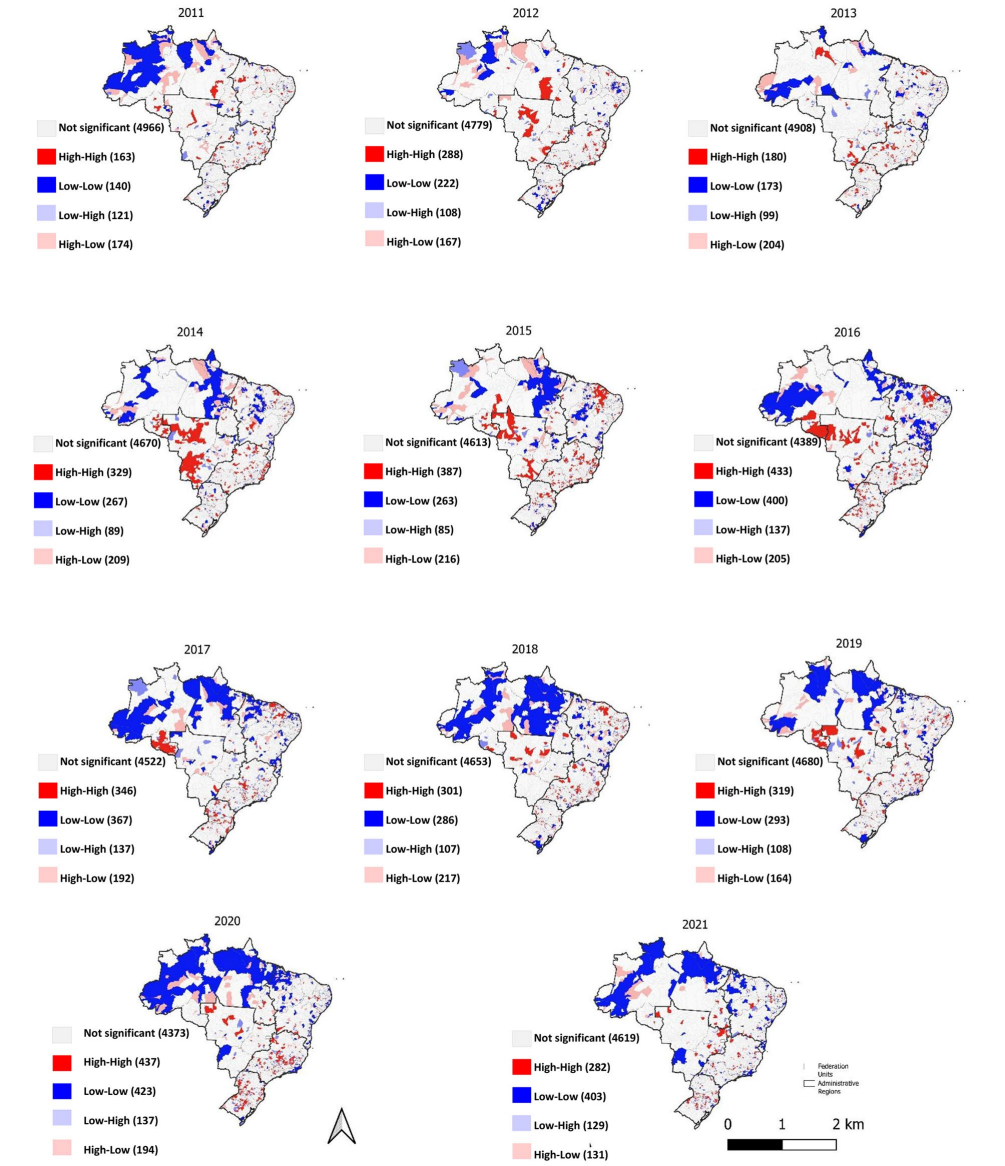
LISA Cluster Map presenting the distribution of statistically significant spatial clusters of polio vaccine coverage in children less than one year of age, Brazil, 2011–2021

In the period from 2011 to 2021, there is a progressive worsening of vaccination coverage in Brazilian municipalities. (Fig. 2). The descriptive spatial analysis of the annual coverage of the polio vaccine – First Booster (first year) of the polio vaccine – Second Booster (fourth year), is presented as supplementary material (Figs. 3S and 4S).

### Spatial analysis

We observed a progressive increase in low-low clusters formed by municipalities with low vaccination coverage surrounded by other municipalities with low vaccination coverage most of which are clusters located in the North and Northeast regions of the country (Fig. 3).

High-high clusters, formed by municipalities with high vaccination coverage rates and surrounded by municipalities that also demonstrated the same behavior, were more frequent in the Midwest, Southeast and South regions during the periods covering 2014 to 2016. From 2017 onwards, the high-high clusters were predominantly located in the Southeast and South regions (Fig. 3). During the periods 2011 to 2016 and 2021, weak spatial autocorrelations were identified, with the Moran’s I from 0.059 to 0.260, while in the period from 2017 to 2020, moderate spatial autocorrelations were also observed. The spatial analysis of coverage of the polio vaccine – First Booster (first year) of the polio vaccine – Second Booster (fourth year) was presented as supplementary material (Figs. 5 and 6 S).

### Trend analysis

The trend analyses suggested that the overall vaccination coverage in Brazil and in the five Regions of the country resulted in decreasing trends in polio vaccine coverage in children less than one year of age (p < 0.05). As for the 26 states and the DF, only the DF, Mato Grosso do Sul and Ceará presented a stationary trend for polio vaccine coverage in children less than one year of age (p > 0.05) (Table 1).

**Table 1:** Trends in polio vaccine coverage in children less than one year of age by Regions and States. National Immunization Program, Brazil, 2011–2021

Variable	p-value	Beta	Standard error	APC	CI* (95%) MIN	CI* (95%) MAX	Tendency
Brazil	**< 0.001**	-0.0144941	0.0024098	-3.28	-4.47	-2.08	**Descending**
Midwest	**0.004**	-0.0135034	0.0034616	-3.06	-4.77	-1.32	**Descending**
Distrito Federal	0.100	-0.0094936	0.0051871	-2.16	-4.73	0.48	**Stationary**
Goiás	**< 0.001**	-0.0168363	0.0019491	-3.80	-4.76	-2.84	**Descending**
Mato Grosso	**< 0.001**	-0.0132844	0.0022782	-3.01	-4.14	-1.87	**Descending**
Mato Grosso do Sul	0.116	-0.0128708	0.0074109	-2.92	-6.54	0.84	**Stationary**
Northeast	**0.003**	-0.0155539	0.0039049	-3.52	-5.43	-1.57	**Descending**
Alagoas	**0.038**	-0.0086579	0.0035534	-1.97	-3.74	-0.17	**Descending**
Bahia	**< 0.001**	-0.0188339	0.0027208	-4.24	-5.57	-2.90	**Descending**
Ceará	0.117	-0.0116937	0.0067500	-2.66	-5.97	0.77	**Stationary**
Maranhão	**< 0.001**	-0.0243640	0.0035292	-5.46	-7.15	-3.73	**Descending**
Paraíba	**0.005**	-0.0148852	0.0040216	-3.37	-5.34	-1.36	**Descending**
Pernambuco	**0.001**	-0.0189313	0.0040422	-4.27	-6.23	-2.26	**Descending**
Piauí	**0.006**	-0.0127025	0.0036016	-2.88	-4.66	-1.07	**Descending**
Rio Grande do Norte	**0.008**	-0.0146934	0.0043629	-3.33	-5.47	-1.14	**Descending**
Sergipe	**< 0.001**	-0.0159123	0.0024582	-3.60	-4.81	-2.37	**Descending**
North	**< 0.001**	-0.0186644	0.0024734	-4.21	-5.41	-2.98	**Descending**
Acre	**0.001**	-0.0202941	0.0041902	-4.57	-6.59	-2.49	**Descending**
Amapá	**0.002**	-0.0312336	0.0070288	-6.94	-10.24	-3.52	**Descending**
Amazonas	**0.013**	-0.0147130	0.0047336	-3.33	-5.65	-0.95	**Descending**
Pará	**0.001**	-0.0253333	0.0048172	-5.67	-7.97	-3.31	**Descending**
Rondônia	**0.038**	-0.0150380	0.0061858	-3.40	-6.42	-0.29	**Descending**
Roraima	**0.033**	-0.0212162	0.0084243	-4.77	-8.80	-0.56	**Descending**
Tocantins	**< 0.001**	-0.0071584	0.0012669	-1.63	-2.27	-0.99	**Descending**
Southeast	**0.002**	-0.0137303	0.0032653	-3.11	-4.72	-1.48	**Descending**
Espírito Santo	**< 0.001**	-0.0138493	0.0015487	-3.14	-3.91	-2.37	**Descending**
Minas Gerais	**0.001**	-0.0102284	0.0019434	-2.33	-3.30	-1.35	**Descending**
Rio de Janeiro	**0.003**	-0.0301750	0.0073272	-6.71	-10.15	-3.14	**Descending**
São Paulo	**< 0.001**	-0.0107252	0.0019775	-2.44	-3.42	-1.44	**Descending**
South	**< 0.001**	-0.0078418	0.0012614	-1.79	-2.42	-1.15	**Descending**
Paraná	**< 0.001**	-0.0093604	0.0014038	-2.13	-2.83	-1.42	**Descending**
Rio Grande do Sul	**< 0.001**	-0.0081588	0.0017614	-1.86	-2.74	-0.97	**Descending**
Santa Catarina	**< 0.001**	-0.0067342	0.0013021	-1.54	-2.19	-0.88	**Descending**

Note: *CI – Confidence interval of Annual Variation (%)

Trend analysis of annual coverage of polio vaccine – First Booster (first year) of Polio vaccine was presented as supplemental material (Table 1 S).

## Discussion

The national target of 95% coverage of the polio vaccine in children under one year of age was achieved by 2014. From 2014 to 2021, coverage progressively declined from 100% to 2014 to 70.1% in 2021. Polio vaccine coverage in children under one year of age in Brazil and the formation of low-low spatial clusters from years 2011 to 2021 accounts for the high number of children susceptible to poliomyelitis, principally in the North and Northeast regions of the country.

In Brazil, a country with the largest population of children under four years of age in Latin America [14], the historic reduction in vaccination coverage, especially from 2016 to present [15, 25], may have been exacerbated by the COVID-19 pandemic contributing to an increase in the number of children who failed to receive the vaccination against polio. Many factors, contextual and individual, contributed to the drop in coverage of vaccines recommended during childhood, among them include social and cultural aspects that affect vaccine acceptance, the introduction of several vaccines to the national vaccination schedule within a short time period, the perception by parents and family members that infectious diseases do not represent a risk for children, since many of these diseases are under control or have been eliminated, and the inconsistency in the availability of immunobiologicals at Primary Care services [15, 18, 29–31].

In this study, we chose municipalities as the spatial unit of analysis because they represented the lowest level of separate data available by the National Immunization Program Information System (SI-NIP) [23]. At the state level, the highest unit of analysis, vaccination coverage is obtained from the average coverage of municipal vaccinations, therefore, may suffer interference from extreme values of vaccination coverage, especially in states that exibit high municipal heterogeneity of vaccination coverage [32]. In this sense, the spatial analysis of vaccination coverage, taking municipalities as units of analysis, can help identify municipalities that demonstrate low vaccination coverage in a prompt or sustained manner during the evaluated period, leading to the accumulation of at-risk children, thus compromising the scope of control or the elimination of a vaccine-preventable infectious disease.

The existence of clusters of municipalities reporting low vaccination coverage surrounded by municipalities also with low vaccination coverage (low-low) demonstrates that this is not an isolated problem across the nation’s cities, highlighting the common difficulties faced by cities and local health departments to achieve vaccine coverage goals as recommended by the NIP. Clusters of municipalities exhibiting low coverage surrounded by municipalities with high coverage (low-high) and clusters of municipalities with high coverage surrounded by municipalities with low coverage are strong candidates for intervention by state and regional policy makers with the intended aim of strengthening the health care network at these locations.

It is noteworthy to report here that the coverage of the polio vaccine in children less than one year of age was not homogeneous across Brazilian states and regions during the evaluated period. The North and Northeast regions, which have the worst social and economic indicators in the country [18, 32], presented the worst indicators of polio vaccine coverage in children less than one year of age during the evaluated study period. Thus, these indicators favored the formation of pockets of individuals susceptible to Poliovirus in these regions. In Brazil, the regional inequality of human development and health indicators is historical [33, 34]. The Southeast, South and Mid-West regions boast a greater Municipal Human Development Index (MHDI) than their neighbors to the North and Northeast regions [34]. The MHDI is an indicator comprised of three dimensions: longevity, education and income, which can vary from 0 to 1. A national study has suggested a trend towards a reduction in polio vaccine coverage in children less than one year of age between 2006 and 2016 and the formation of clusters by municipalities with low vaccination coverage located in the North and Northeast regions, corroborating our results and reinforcing the historical discrepancy in vaccination coverage between the five regions of Brazil [18, 19].

Furthermore, Northern regional states of the country have some particularities, among them, are inhabitants which reside along riverbanks and who require small wooden boats for mobility and must navigate average distances of to 60 km, for 4 h, to access the nearest health care facilities [35]. The States of Acre and Amazonas, both located in the North Region, have the largest riverside populations in Brazil [36]. It is also noteworthy that some municipalities in these states have territorial extension that surpass some Brazilian states, with the concentration of health services being observed in municipalities that are on the banks of large rivers, leaving the population that lives far from them uncovered [37]. It is also noteworthy that some municipalities in these states have territorial extension that surpass some Brazilian states, with the concentration of health services being observed in municipalities that are on the banks of large rivers, leaving the population that lives far from them uncovered [35, 38]. In addition to the geographical barriers, the extensive cross-border territory also poses challenges and, since 2016, the Northern region has received approximately 260,000 Venezuelan refugees who entered Brazil through the city of Pacaraíma, the Venezuelan-Brazilian border located in the Brazilian state of Roraima [39]. The political and economic instability in Venezuela has compromised the population’s access to employment, health services and housing, triggering the migratory flow of Venezuelans to Latin American countries, especially to Brazil [39].

It is also worth noting that, during the COVID-19 pandemic, immunization strategies against poliomyelitis were interrupted in these countries, especially those immunization strategies that were done door-to-door [6, 7]. This interruption, even for a short period of time, has compromised polio vaccine coverage and represents a barrier to achieving a Polio Free World by 2026, a target set out in the Polio Eradication Strategy 2022–2026 [6, 7].

In this context, migratory movements, combined with low vaccination coverage, civil war, economic and political instability in Pakistan and Afghanistan, the last bastions of the WP1 strain in the world [6], can contribute to the spread of Poliovirus to the rest of the world, affecting countries with low vaccination coverage and high risk of cVDPV emergence or importation of Poliovirus, among them, Brazil.

### Limitations

Some limitations of this study must be considered. First, this study was subject to information bias, since the researchers did not have access to quality-controlled SI-NIP information records. However, the SI-NIP constitutes an important official source of information. Coordinated by the Ministry of Health, the SI-NIP is technically solid and, over the last 40 years, has allowed the monitoring of vaccination coverage and provides technical assistance at the federal, state and municipal levels for guidance and decision-making necessary for the best choose health strategy practices aimed at preventing infectious diseases [15]. Second, considering that in this study the coverage of vaccination against poliomyelitis according to a birth cohort was not evaluated, the results may be subject to the influences of the context, lifestyles and behaviors from that period, since the data were analyzed over a single, historic moment. The cohort design, although it has several advantages, adequately considers children who were vaccinated in a timely manner (up to 5 years of age). Considering the importance of the immune status and the production of antibodies against the Poliovirus in one-year old children, due to the greater risk of a severe sequelae caused by the Poliovirus, the authors decide to continue with a cross-sectional design, considering as adequately vaccinated, those children who had received their scheduled vaccinations at one year of age. In addition, this study was done with the entire target population of the polio vaccine in children under one year of age, using the SI-NIP, which is considered a reliable official source of vaccine coverage data in Brazil, with generalizations taken as relatively safe estimates. Another limitation of this study is that vaccination coverage was obtained using the indirect method, in which the administered doses of the vaccines were obtained from more than one data source and depend on the quality of the registration of doses administered by the health professional.

### Contributions

This study may contribute to the identification of territories that presented unsatisfactory indicators on vaccination coverage targeting poliomyelitis in Brazil, which highlights the urgent need to prioritize vaccination strategies and efforts at increasing coverage to vulnerable populations from these regions. In addition to the territories that reported vaccine coverage against poliomyelitis below the 95% target, those with a sharp drop in vaccination coverage in the evaluated period should also be prioritized. In this sense, vaccination strategies that address both the resident inhabitants and the population of travelers, migrants and refugees should be given full priority in these territories.

## Conclusion

Over the period spanning 2011 to 2021, there was an observed downward trend in polio vaccine coverage in eight Brazilian states and a progressive increase in spatial clusters with low vaccine coverage located particularly in the North and Northeast regions of the country. Immunization strategies and public policies aimed at reducing inequalities of vaccination coverage in the country are essential to halt the preventable burden of polio and achieve the goal of immunization in relation to the 2030 Agenda for Sustainable Global Goals. Furthermore, improvement of polio vaccine coverage indicators is essential to prevent the reintroduction of Poliovirus in Brazil.

## Electronic supplementary material

Below is the link to the electronic supplementary material.

Supplementary Material 1

## Data Availability

The datadase used and/or analysed during the current study available at http://tabnet.datasus.gov.br/cgi/dhdat.exe?bd_pni/cpnibr.def.

## Abbreviations

cVDPV: Circulating vaccine-derived poliovirus
VDPV: Vaccine-derived poliovirus
IPV: Inactivated Polio Vaccine
OPV: Oral Polio Vaccine
cVDPV2: Type 2 Circulating Vaccine-Derived Poliovirus
WHO: World Health Organization
WPV1: Wild Poliovirus, type 1
WPV2: Wild Poliovirus, type 2
WPV3: Wild Poliovirus, type 3
GPEI: Global Polio Eradication Initiative
I_ML_: Moran’s I
NIP: National Immunization Program
CI: Confidence Interval (%)
SI-NIP: Brazilian National Immunization Information Program System
95% CI: The 95% Confidence Intervals
APC: Annual Percent Change
ep: Standard Error
Min: Minimum
Max: Maximum

## References

[1] Verani JFS, Laender F. Poliomyelitis eradication in four stages. Cad Saude Publica. 2020 Nov 2;36(Suppl 2):e00145720. 10.1590/0102-311X00145720. 10.1590/0102-311X00145720 33146314

[2] Patel M, Cochi S (2017). Addressing the Challenges and Opportunities of the Polio Endgame: Lessons for the future. J Infect Dis.

[3] Martinez-Bakker M, King AA, Rohani P (2015). Unraveling the transmission ecology of polio. PLoS Biol.

[4] Mehndiratta MM, Mehndiratta P, Pande R, Poliomyelitis (2014). Historical facts, epidemiology, and current Challenges in Eradication. The Neurohospitalist.

[5] Waheed Y (2018). Polio eradication challenges in Pakistan. Clin Microbiol Infect.

[6] Haqqi A, Zahoor S, Aftab MN, Tipu I, Rehman Y, Ahmed H (2021). COVID-19 in Pakistan: impact on global polio eradication initiative. J Med Virol.

[7] World Health Organization, Global Polio Eradication Initiative (GPEI) (2021). Polio Eradication Strategy 2022–2026: delivering on a promise.

[8] WHO. Statement of the Twenty-Ninth Polio IHR Emergency Committee. https://www.who.int/news/item/20-08-2021-statement-of-the-twenty-ninth-polio-ihr-emergency-committee (accessed Dec 10, 2022).

[9] Modlin JF, Bandyopadhyay AS, Sutter R (2021). Immunization against Poliomyelitis and the Challenges to Worldwide Poliomyelitis Eradication. J Infect Dis.

[10] World Health Organization (2022). Global Polio Eradication Initiative Investment Case 2022–2026: investing in the promise of a polio-free world.

[11] WHO. Statement of the Twenty-Ninth Polio IHR Emergency Committee. https://www.who.int/news/item/12-05-2023-statement-of-the-thirty-fifth-polio-ihr-emergency-committee (accessed Jun 10, 2023).

[12] WHO. Statement of the Thirty-second Polio IHR Emergency Committee. https://www.who.int/news/item/24-06-2022-statement-of-the-thirty-second-polio-ihr-emergency-committee (accessed Dec 10, 2022).

[13] Pan American Health Organization (2021). XXVI Meeting of PAHO’s Technical Advisory Group (TAG) on vaccine-preventable Diseases.

[14] da Silva TMR, de Sá ACMGN, Vieira EWR, Prates EJS, Beinner MA, Matozinhos FP (2021). Number of doses of Measles-Mumps-Rubella vaccine applied in Brazil before and during the COVID-19 pandemic. BMC Infect Dis.

[15] Domingues CMAS, Maranhão AGK, Teixeira AM, Fantinato FFS, Domingues RAS. The Brazilian National Immunization Program: 46 years of achievements and challenges. Cad Saude Publica. 2020 Oct 26;36(Suppl 2):e00222919. 10.1590/0102-311X00222919. 10.1590/0102-311X00222919 33111749

[16] Buffarini R, Barros FC, Silveira MF (2020). Vaccine coverage within the first year of life and associated factors with incomplete immunization in a brazilian birth cohort. Arch Public Heal.

[17] Césare N, Mota TF, Lopes FFL, Lima ACM, Luzardo R, Quintanilha LF (2020). Longitudinal profiling of the vaccination coverage in Brazil reveals a recent change in the patterns hallmarked by differential reduction across regions. Int J Infect Dis.

[18] Silveira MF, Tonial CT, Goretti K, Maranhão A, Teixeira AMS, Hallal PC, Maria B, Menezes A (2021). Missed childhood immunizations during the COVID-19 pandemic in Brazil: analyses of routine statistics and of a national household survey. Vaccine.

[19] Arroyo LH, Ramos ACV, Yamamura M, Weiller TH, Crispim J, de Cartagena-Ramos A (2020). [Areas with declining vaccination coverage for BCG, poliomyelitis, and MMR in Brazil (2006–2016): maps of regional heterogeneity]. TT - Áreas com queda da cobertura vacinal para BCG, poliomielite e tríplice viral no Brasil (2006–2016): mapas da heterogen. Cad Saude Publica.

[20] World Health Organization (2020). Immunization agenda 2030: a global strategy to leave no one behind. World Heal Organ.

[21] Silva TMR, Da, Nogueira de Sá ACMG, Beinner MA, Abreu MNS, Matozinhos FP, Sato APS (2022). Impact of the COVID-19 pandemic on human papillomavirus vaccination in Brazil. Int J Public Health.

[22] Barros AJD, Santos I, da dos S, Victora CG, Albernaz EP, Domingues MR, Timm IK (2006). Coorte de nascimentos de Pelotas, 2004: metodologia e descrição. Rev Saúde Pública.

[23] Brasil M, Saúde (2022). Secretaria de Vigilância em Saúde, Departamento de Articulação Estratégica, de Vigilância em Saúde. Guia de Vigilância em Saúde.

[24] Instituto Brasileiro de Geografia e Estatística. Cidades e Estados. https://www.ibge.gov.br/cidades-e-estados (accessed Jun 10, 2023).

[25] Pacheco FC, França GVA, Elidio GA, Domingues CMAS, de Oliveira C, Guilhem DB (2019). Trends and spatial distribution of MMR vaccine coverage in Brazil during 2007–2017. Vaccine.

[26] Anselin L, Local Indicators of Spatial Association-LISA (2010). Geogr Anal.

[27] Anselin L, Syabri I, Kho Y, GeoDa (2006). An introduction to spatial data analysis. Geogr Anal.

[28] Antunes JLF, Cardoso MRA (2015). Uso da análise de séries temporais em estudos epidemiológicos. Epidemiol e Serviços Saúde.

[29] Vieira EW, Pimenta AM, Montenegro LC, Silva TMR da. Structure and location of vaccination services influence the availability of the triple viral in Brazil. Reme Rev Min Enferm 2020;24:1–6. 10.5935/1415-2762.20200062.

[30] Sato APS. What is the importance of vaccine hesitancy in the drop of vaccination coverage in Brazil? Rev Saude Publica. 2018 Nov 29;52:96. 10.11606/S1518-8787.2018052001199. 10.11606/S1518-8787.2018052001199 PMC6284490 30517523

[31] De Oliveira VC, De Azevedo Guimarães EA, Perez G, Zacharias FCMH, Cavalcante RB, Gontijo TL (2020). Factors related to the adoption of the brazilian National Immunization Program Information System. BMC Health Serv Res.

[32] Tauil M, de Sato C, Waldman APS (2016). Factors associated with incomplete or delayed vaccination across countries: a systematic review. Vaccine.

[33] Mullachery P, Silver D, Macinko J. Changes in health care inequity in Brazil between 2008 and 2013. Int J Equity Health. 2016 Nov 17;15(1):140. 10.1186/s12939-016-0431-8. 10.1186/s12939-016-0431-8 PMC5112635 27852309

[34] Programa das Nações Unidas para o Desenvolvimento. Desenvolvimento humano nas macrorregiões brasileiras. Brasília; 2016. https://repositorio.ipea.gov.br/bitstream/11058/6217/1/Desenvolvimento%20humano%20nas%20macrorregi%C3%B5es%20brasileiras.pdf (accessed Dec 10, 2022).

[35] Guimarães AF, Barbosa VLM, da Silva MP, Portugal JKA, Reis MHS, Gama ASM. Access to health services for riverside residents in a municipality in Amazonas State, Brazil. Rev Pan-Amazônica Saúde 2020;11. 10.5123/S2176-6223202000178.

[36] Gama ASM, Fernandes TG, Parente RCP, Secoli SR. Inquérito de saúde em comunidades ribeirinhas do Amazonas, Brasil. Cad Saude Publica. 2018 Feb 19;34(2):e00002817. 10.1590/0102-311X00002817. 10.1590/0102-311X00002817 29489939

[37] Garnelo L, Lima JG, Soares E, Rocha C, Herkrath FJ (2018). Acesso e cobertura da Atenção Primária à Saúde para populações rurais e urbanas na região norte do Brasil. Saúde em Debate.

[38] Carneiro VCCB, Oliveira PTR, Carneiro SR, Maciel MC, Pedroso JDS. Impact of expansion of primary care in child health: a population-based panel study in municipalities in the Brazilian Amazon. BMJ Open. 2022 Mar 4;12(3):e048897. 10.1136/bmjopen-2021-048897. 10.1136/bmjopen-2021-048897 PMC8900036 35246414

[39] Human Trafficking and Migrant Smuggling Section (UNODC) (2021). Daya Hayakawa Almeida. Relatório Situacional Brasil: TRÁFICO DE PESSOAS EM FLUXOS MIGRATÓRIOS MISTOS, EM ESPECIAL DE VENEZUELANOS.

