# Supplementary material for: Temporal and spatial distribution trends of polio vaccine coverage in less than one-year old children in Brazil, 2011–2021
Source: BMC Public Health. 2023 Jul 14;23:1359. doi: 10.1186/s12889-023-16192-8 (PMC10349464; doi:10.1186/s12889-023-16192-8)
Supplement: Supplementary file 1 — Supplementary Material 1 [file 12889_2023_16192_MOESM1_ESM.docx]

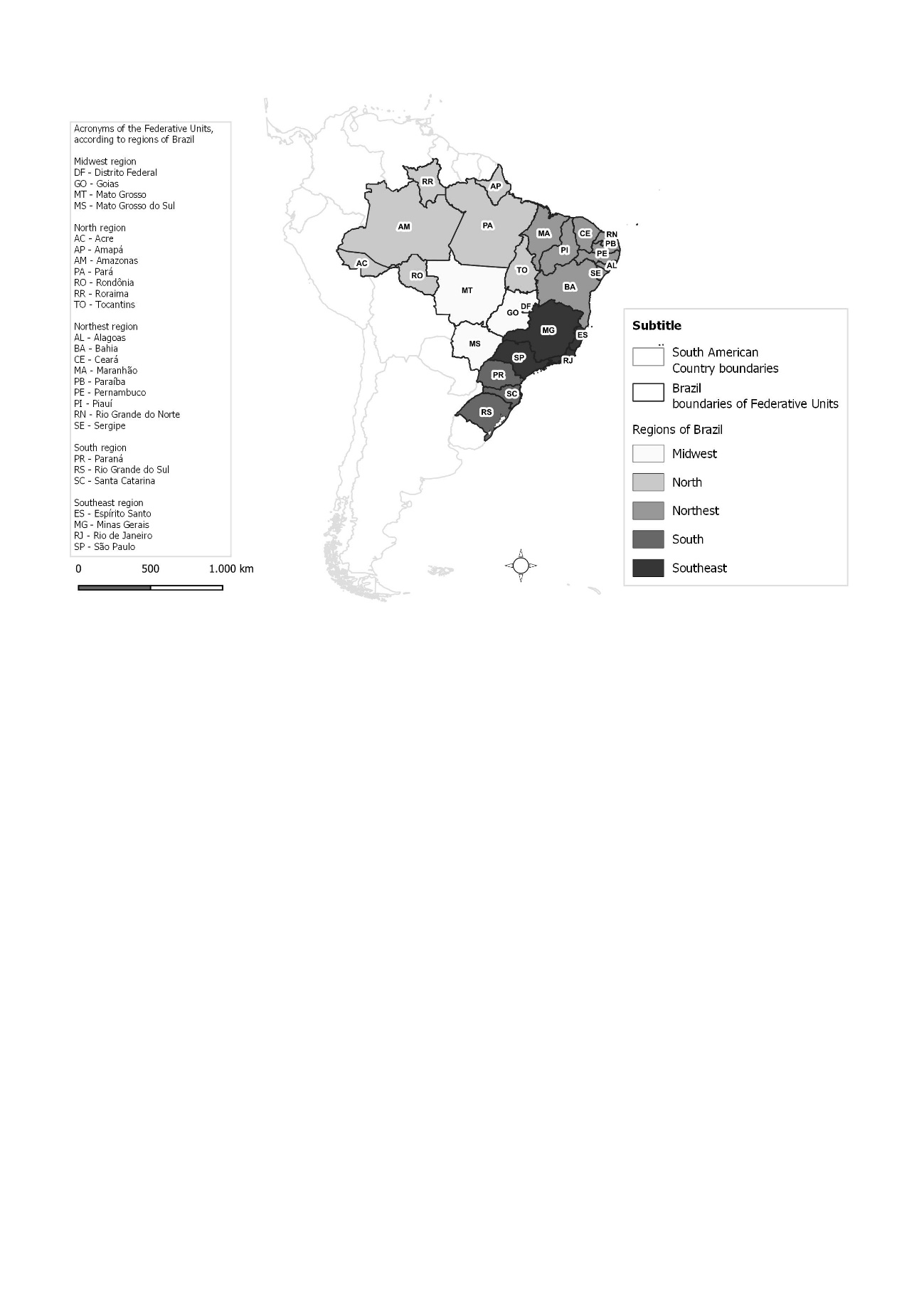


Figure 1S - Map of Brazil divided according to five regions and 27 states. Brazilian Institute of Geography and Statistics, 2022.

| **Year** | **Polio vaccine coverage in children less than one year-old**  (Vaccination coverage in children aged less than or equal to 1 year = number of last doses of the vaccination schedule, applied to children aged less than or equal to 1 year, in a given place and period / number of live births made available at Sinasc for the respective place and period X 100) | **Coverage of the first Polio Vaccine booster (1 ano de idade)**  (Vaccination coverage in children aged 1 year = number of last doses of the vaccination schedule, applied to the population aged one year, in a given location and period / Population aged one year, in a given location, estimated by Brazilian Institute of Geography and Statistics – IBGE, for the last year of period X 100) | **Coverage of the second booster of the Polio Vaccine (4 anos)**  (Vaccination coverage in 4-year-old children = number of last doses of the vaccination schedule, applied to the 4-year-old population, in a given place and period / 4-year-old population, in a given place and period, estimated by Brazilian Institute of Geography and Statistics -IBGE, for the last year of period X 100) |
| --- | --- | --- | --- |
| **2011** | **x** |  |  |
| **2012** | **x** |  |  |
| **2013** | **x** | **x** |  |
| **2014** | **x** | **x** |  |
| **2015** | **x** | **x** |  |
| **2016** | **x** | **x** |  |
| **2017** | **x** | **x** |  |
| **2018** | **x** | **x** | **x** |
| **2019** | **x** | **x** | **x** |
| **2020** | **x** | **x** | **x** |
| **2021** | **x** | **x** | **x** |

Figure 2S- Formulas for calculating polio vaccination coverage by year and age group.

Source: National Immunization Program, Brazil.


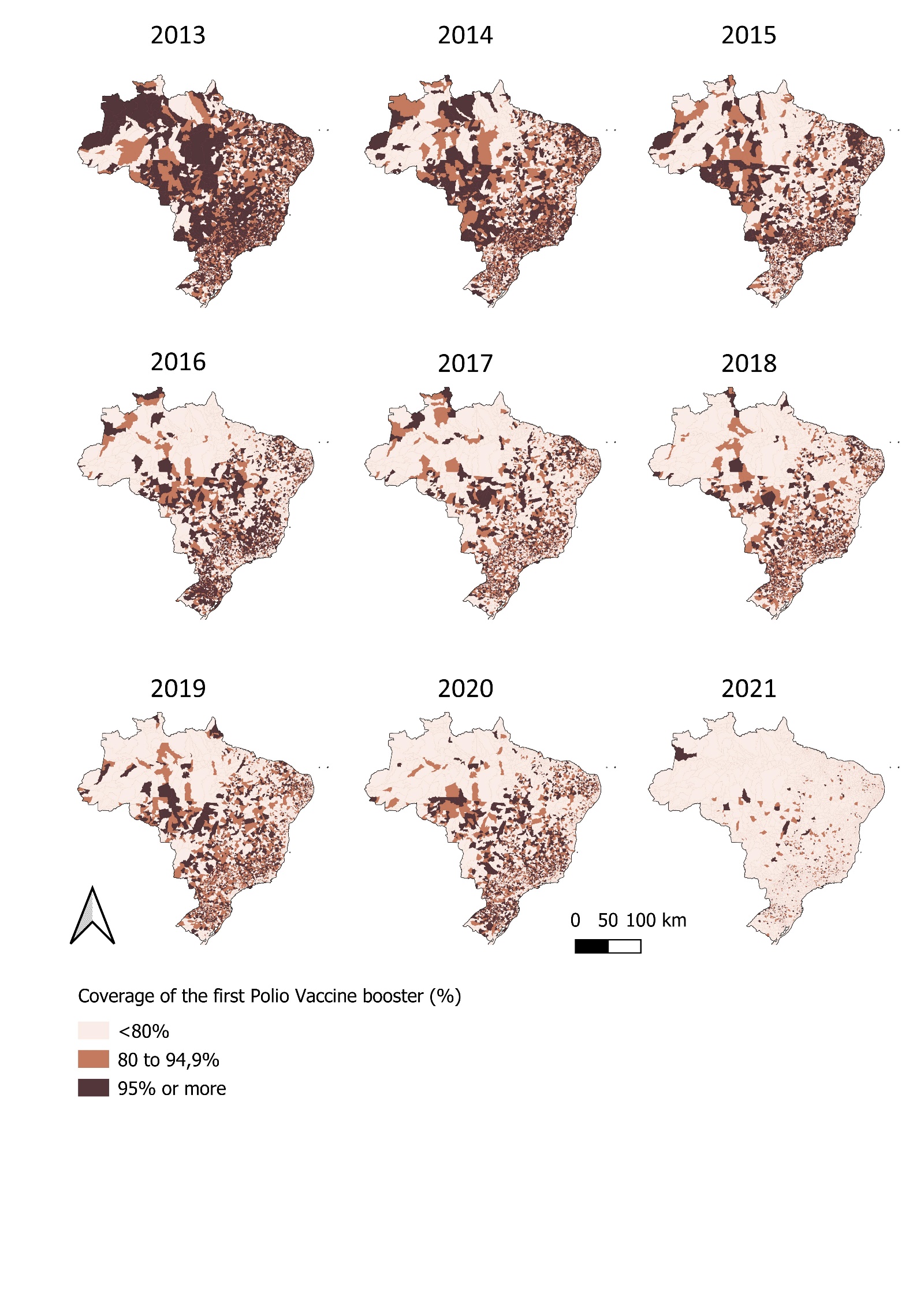


Figure 3S - Annual evolution of the annual coverage (%) of the polio vaccine – First Booster (first year), according to the coverage strata, by states, 2011–2021.


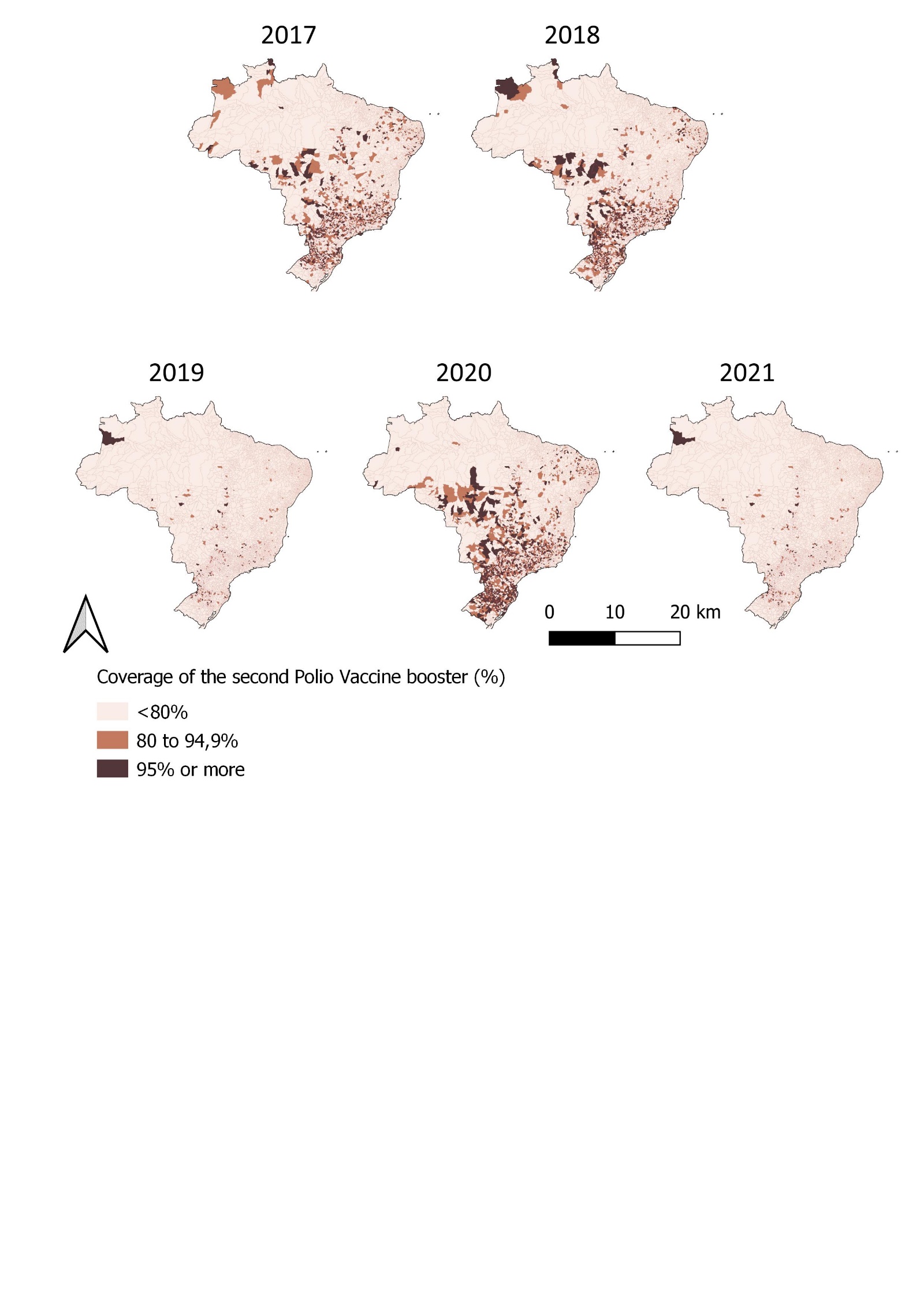


Figure 4S - Annual evolution of the annual coverage of the polio vaccine – Second Booster (fourth year), according to the coverage strata, by states, 2011–2021.


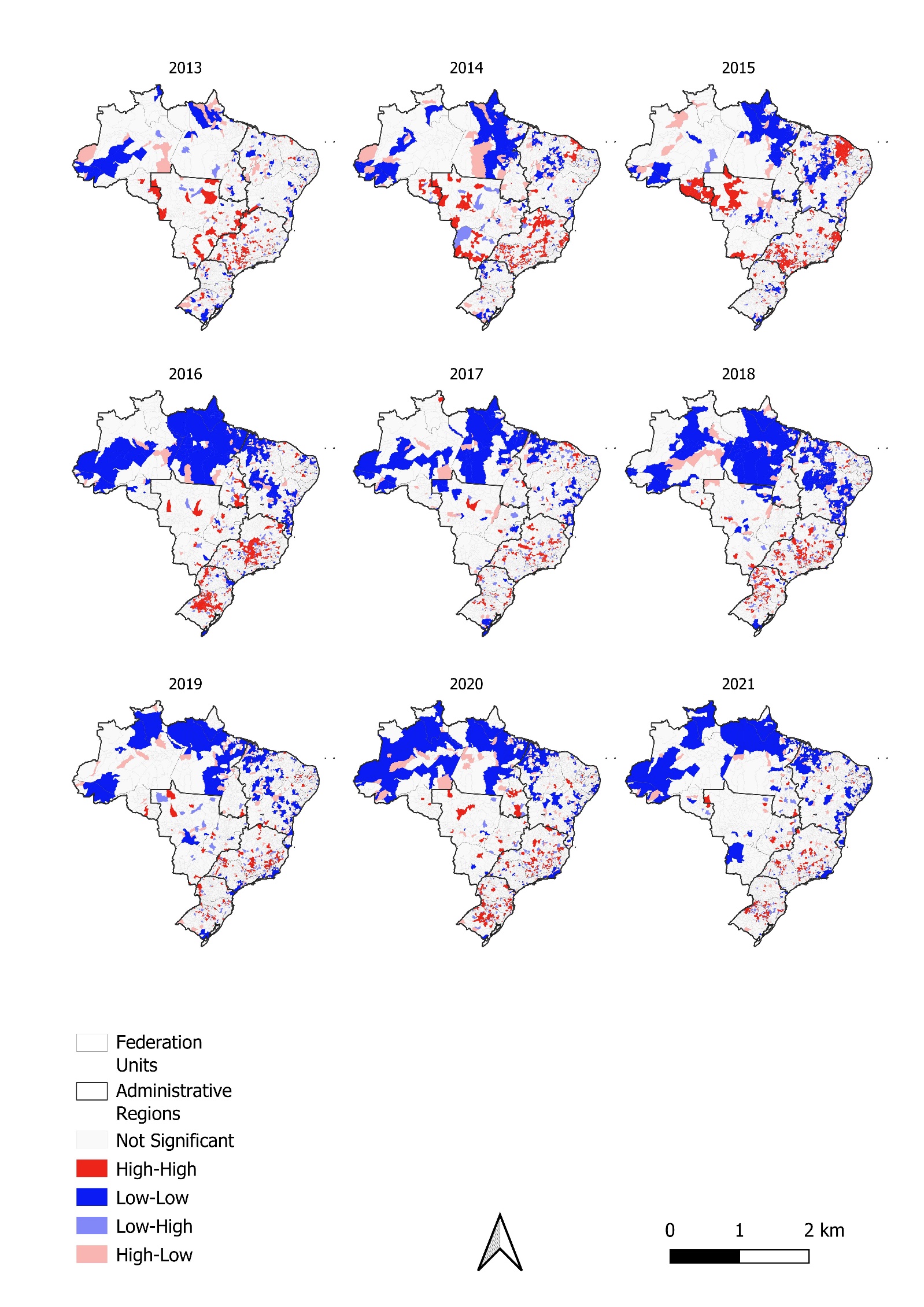


Moran’s I = 0,109


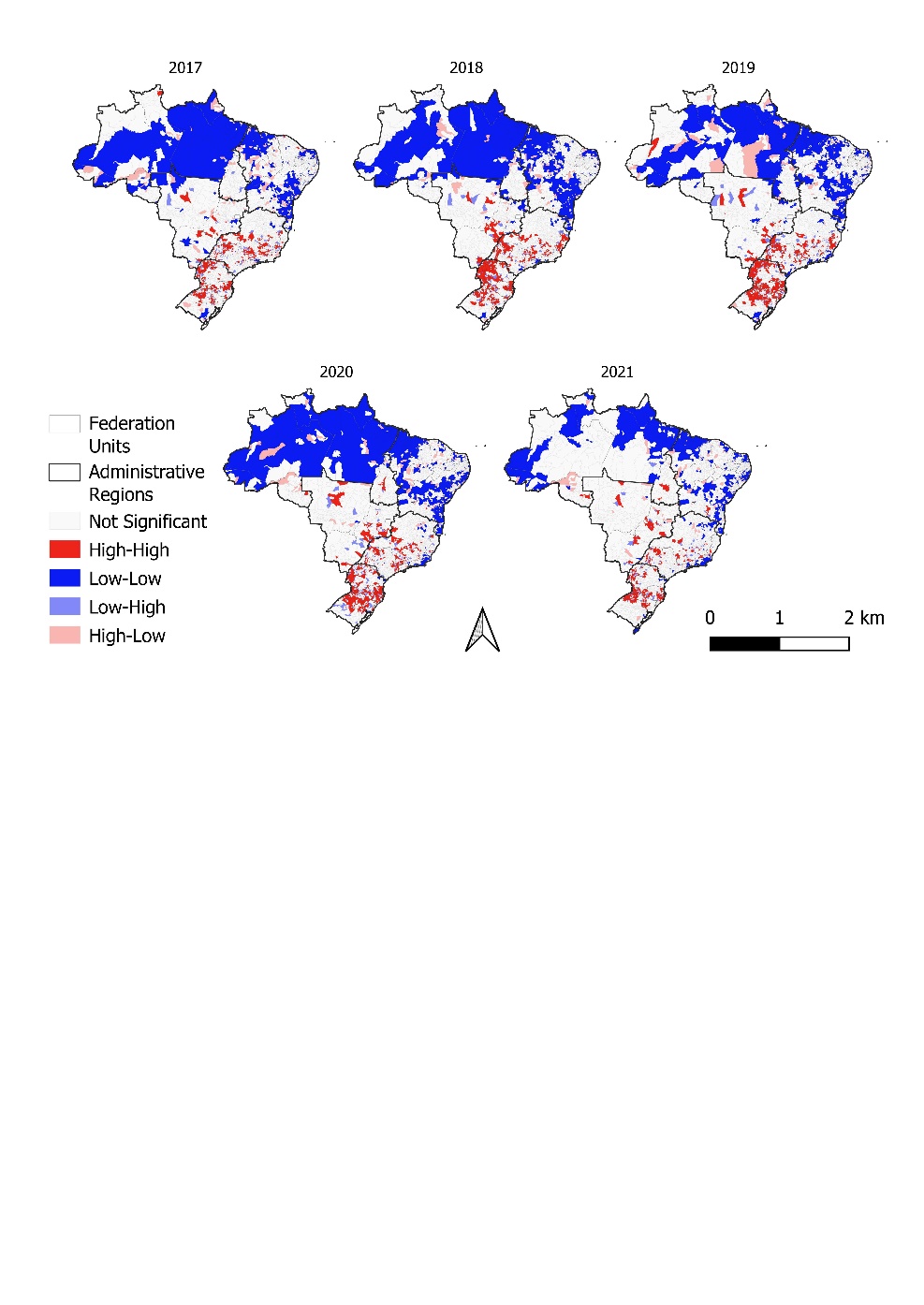

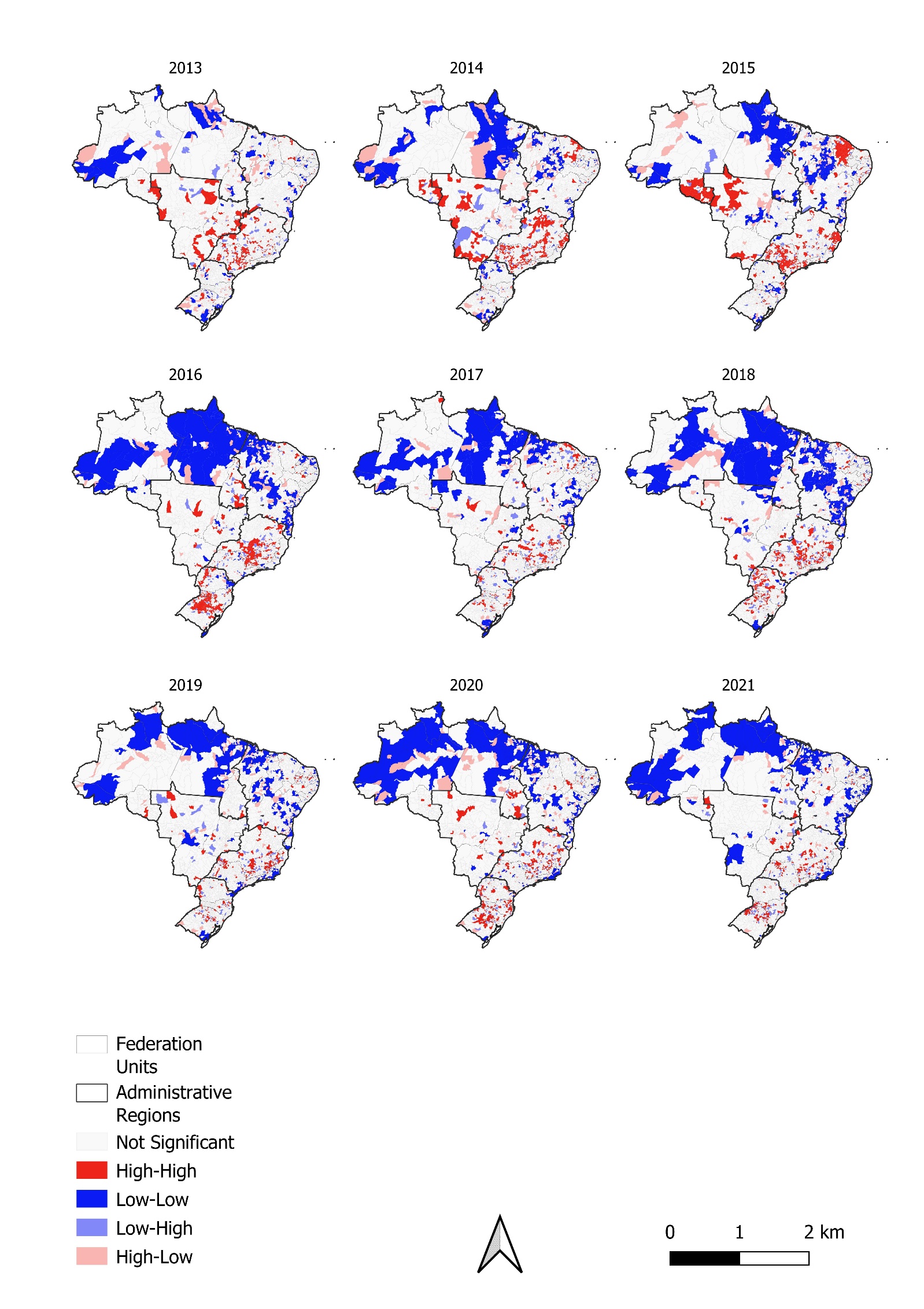

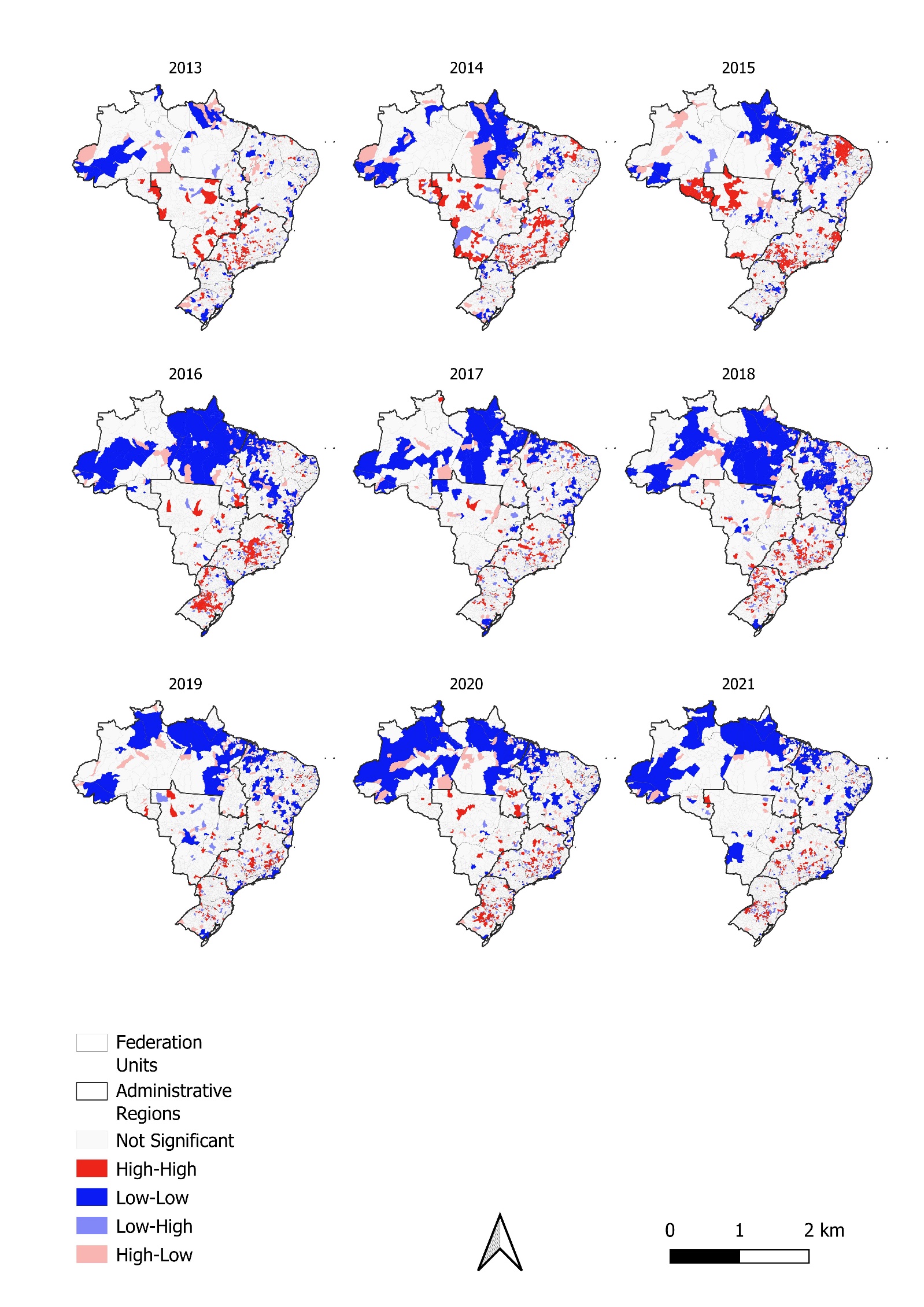

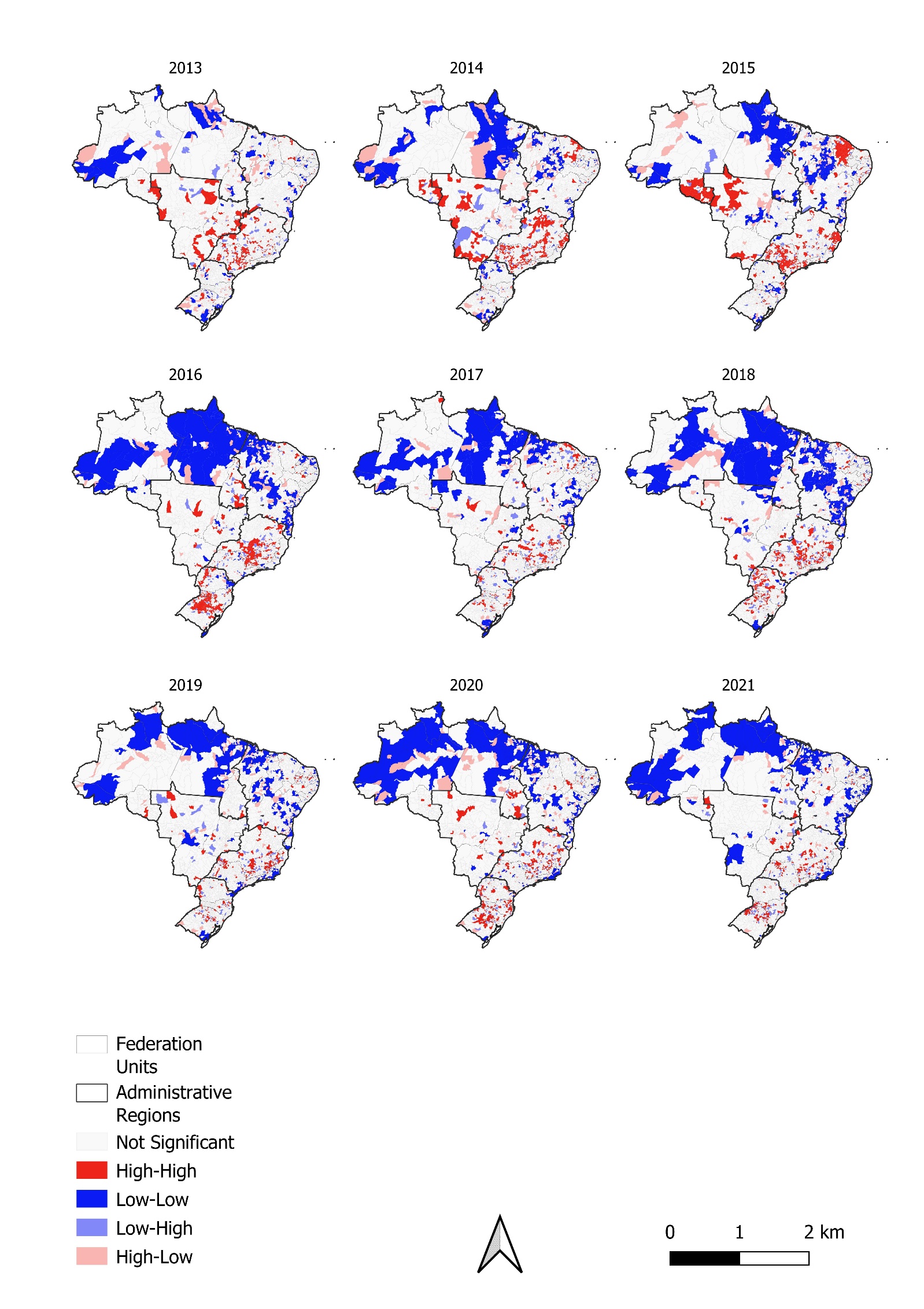

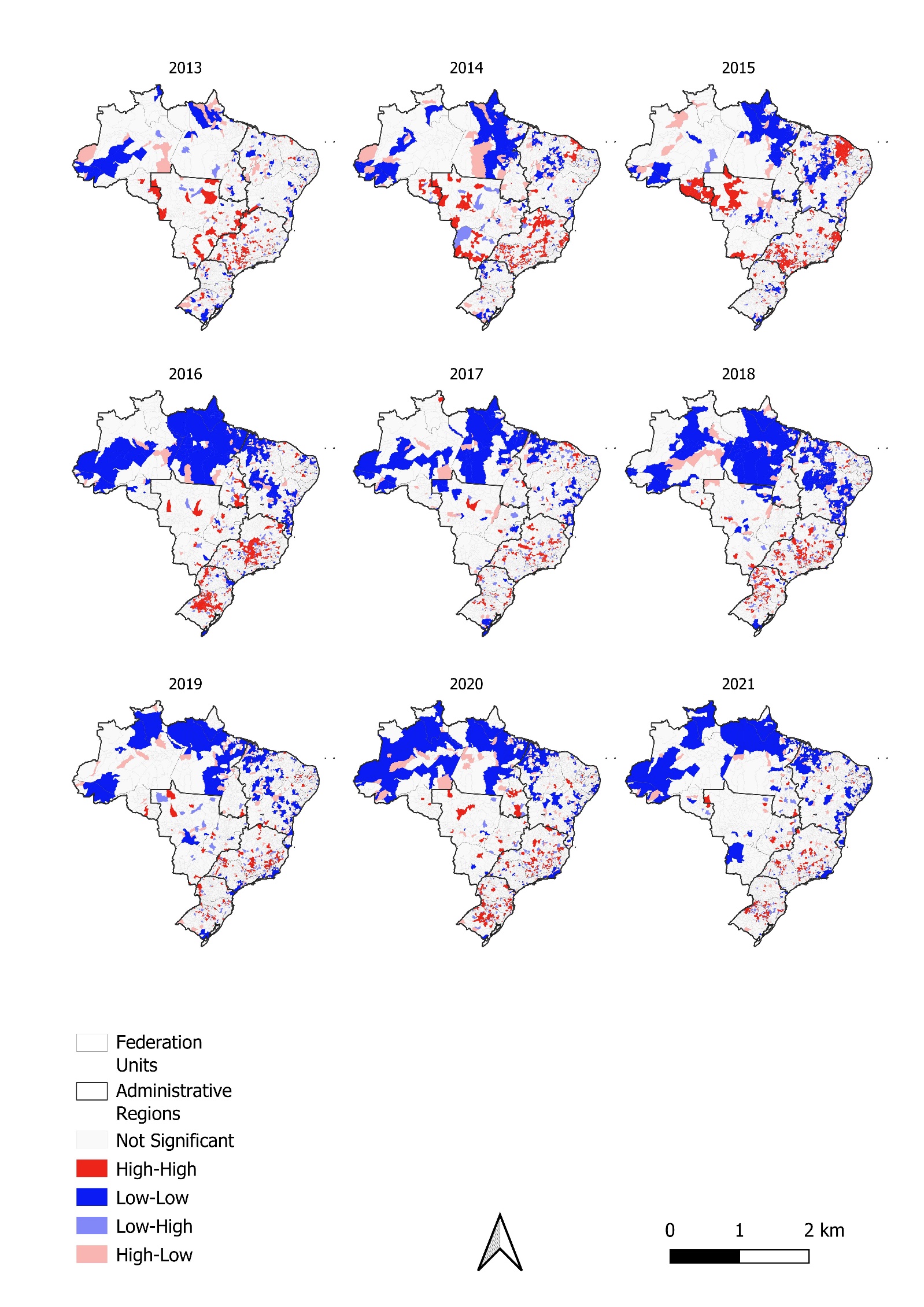

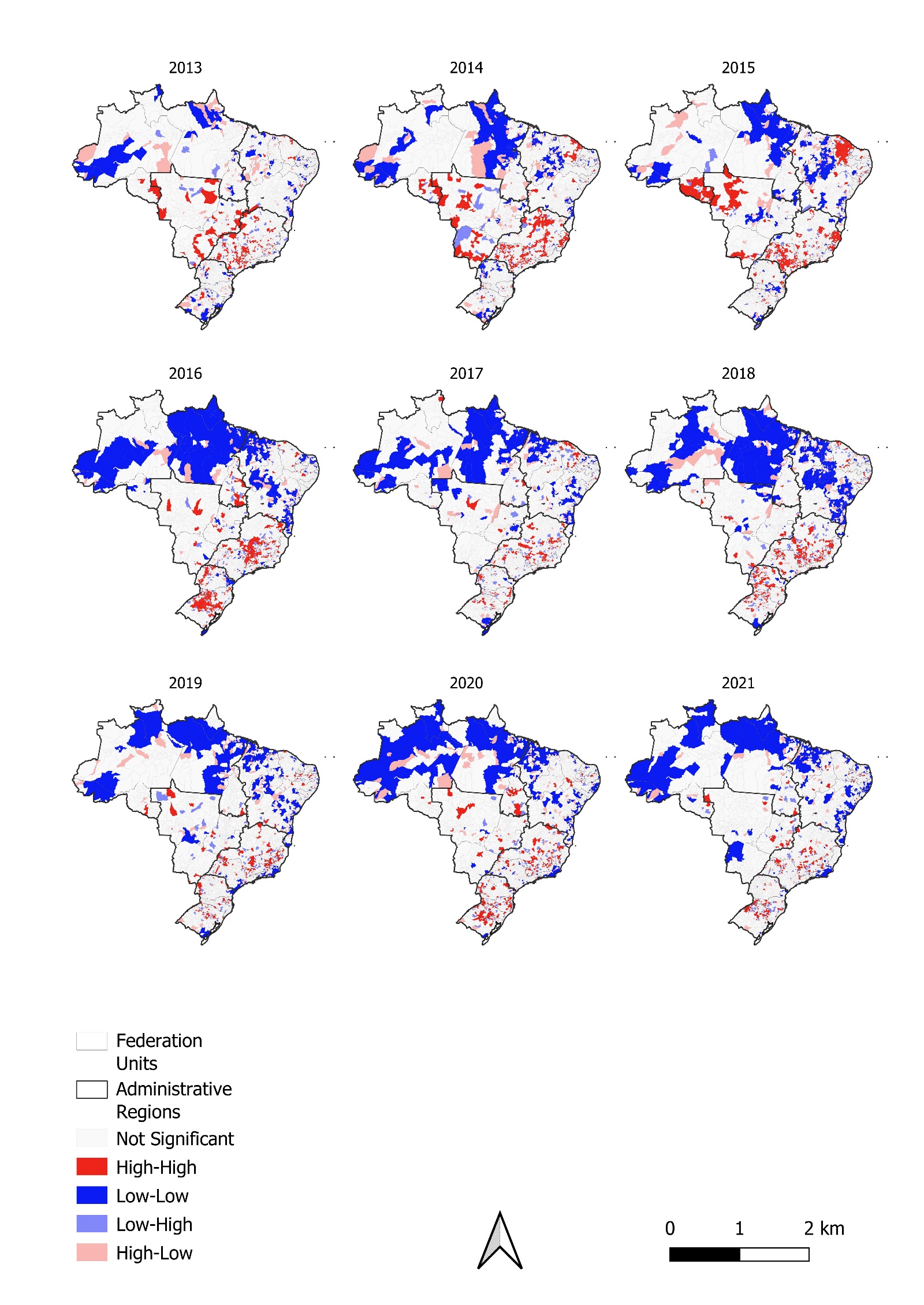

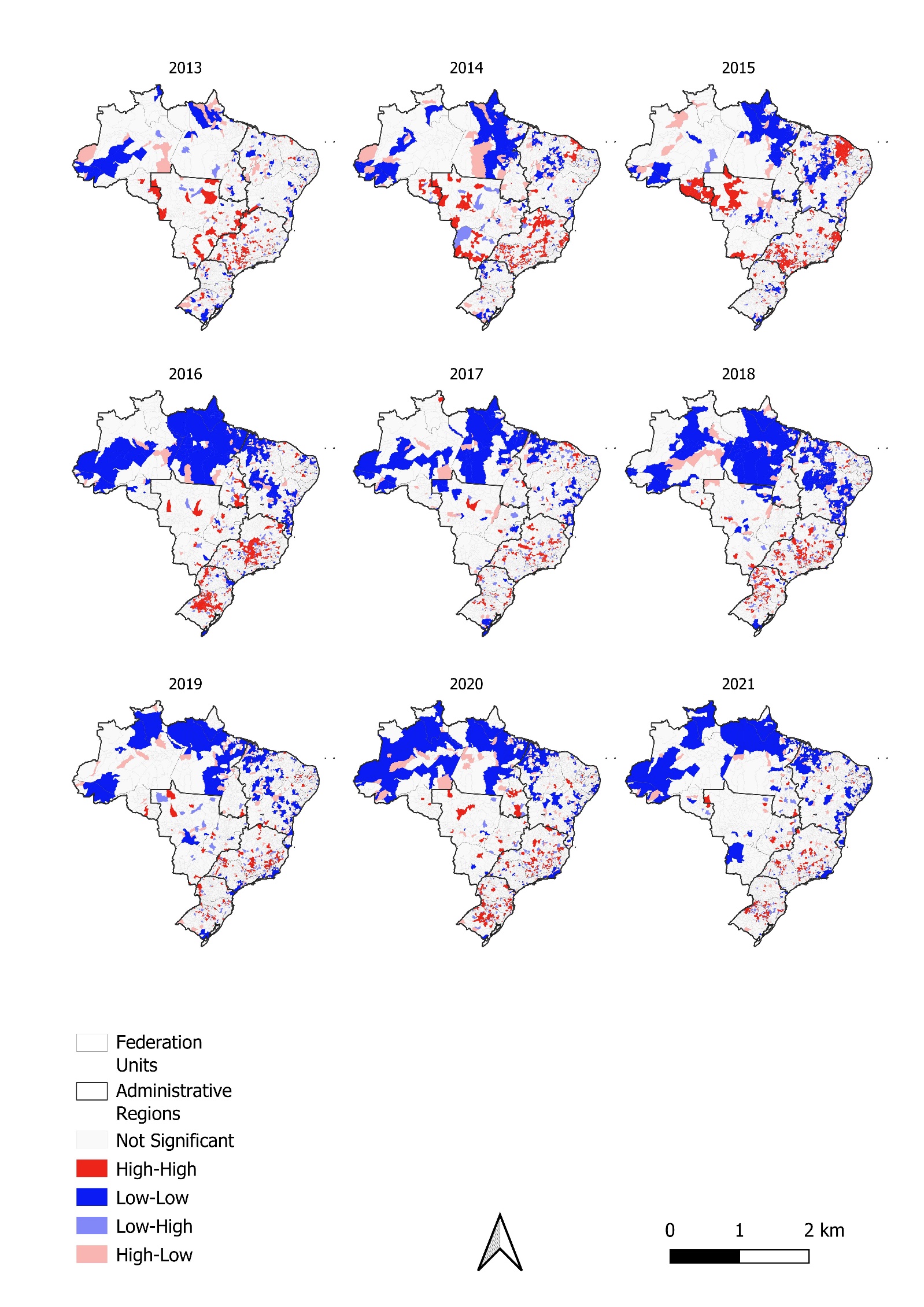

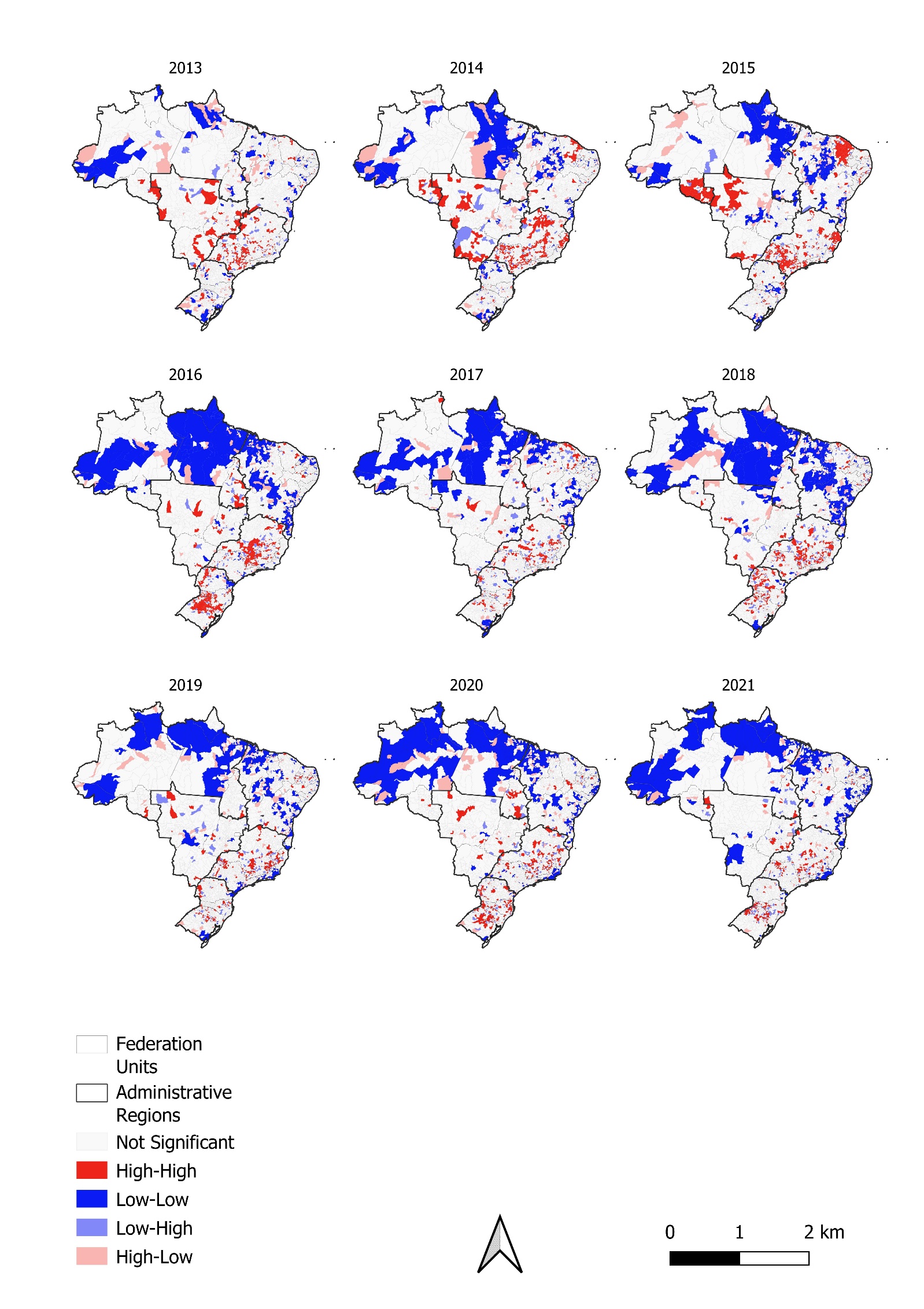

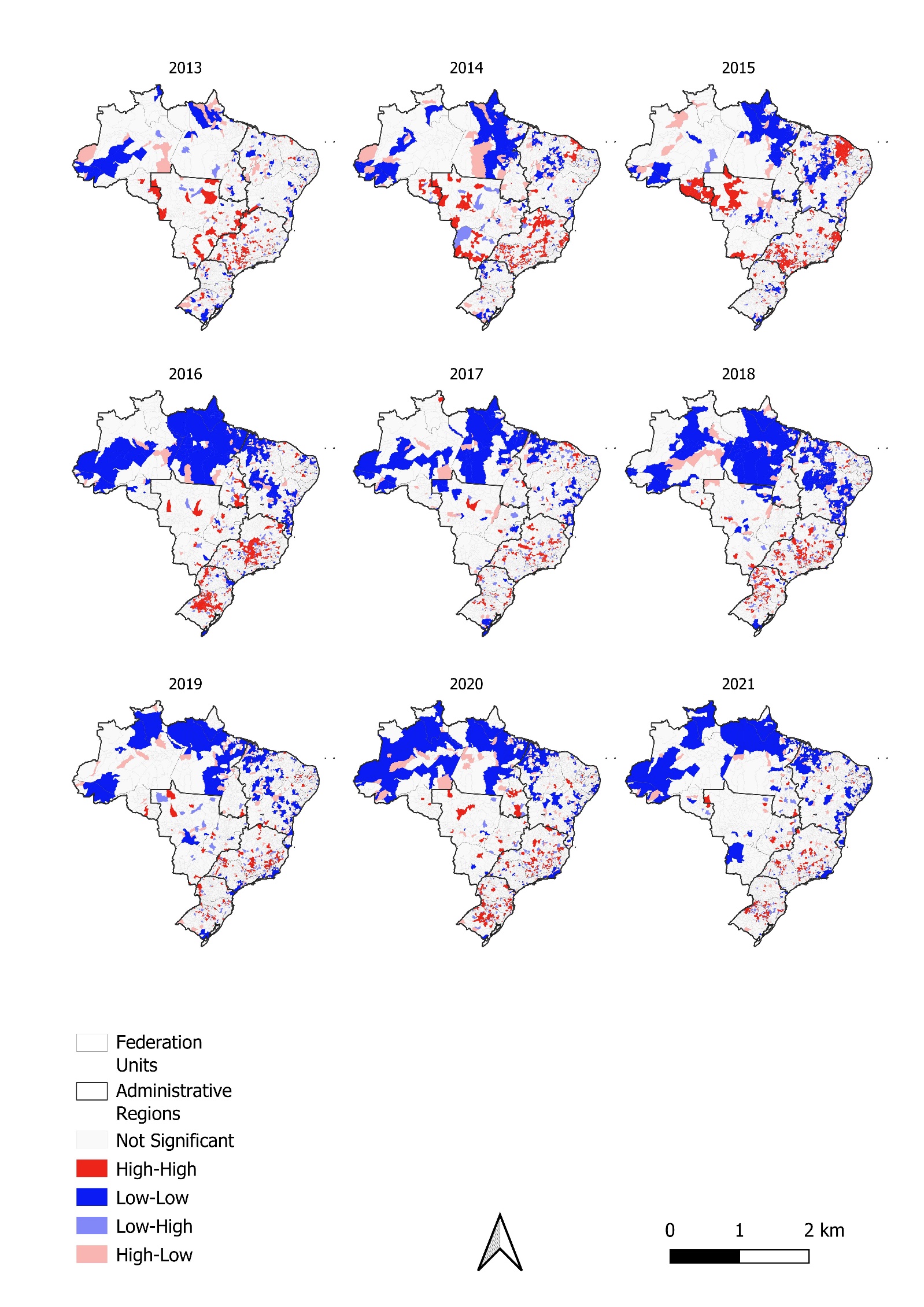


**Not significant (4189)**

**High-High (498)**

**Low-Low (540)**

**Low-High (145)**

**High-Low (192)**

**Not significant (4683)**

**High-High (360)**

**Low-Low (237)**

**Low-High (98)**

**High-Low (186)**

**Not significant (4390)**

**High-High (495)**

**Low-Low (376)**

**Low-High (117)**

**High-Low (186)**

**Not significant (4237)**

**High-High (617)**

**Low-Low (433)**

**Low-High (95)**

**High-Low (182)**

**Not significant (4047)**

**High-High (630)**

**Low-Low (572)**

**Low-High (133)**

**High-Low (182)**

**Not significant (4487)**

**High-High (344)**

**Low-Low (413)**

**Low-High (156)**

**High-Low (164)**

**Not significant (4070)**

**High-High (515)**

**Low-Low (646)**

**Low-High (167)**

**High-Low (166)**

**Not significant (4359)**

**High-High (391)**

**Low-Low (473)**

**Low-High (158)**

**High-Low (183)**

**Not significant (4336)**

**High-High (366)**

**Low-Low (586)**

**Low-High (156)**

**High-Low (138)**

Moran’s I = 0,234

Moran’s I = 0,261

Moran’s I = 0,213

Moran’s I = 0,190

Moran’s I = 191

Moran’s I = 0,204

Moran’s I = 0,189

Moran’s I = 0,267

Figure 5S - LISA Cluster Map presenting the distribution of statistically significant spatial clusters of the polio vaccine coverage – First Booster (first year), Brazil, 2013–2021.


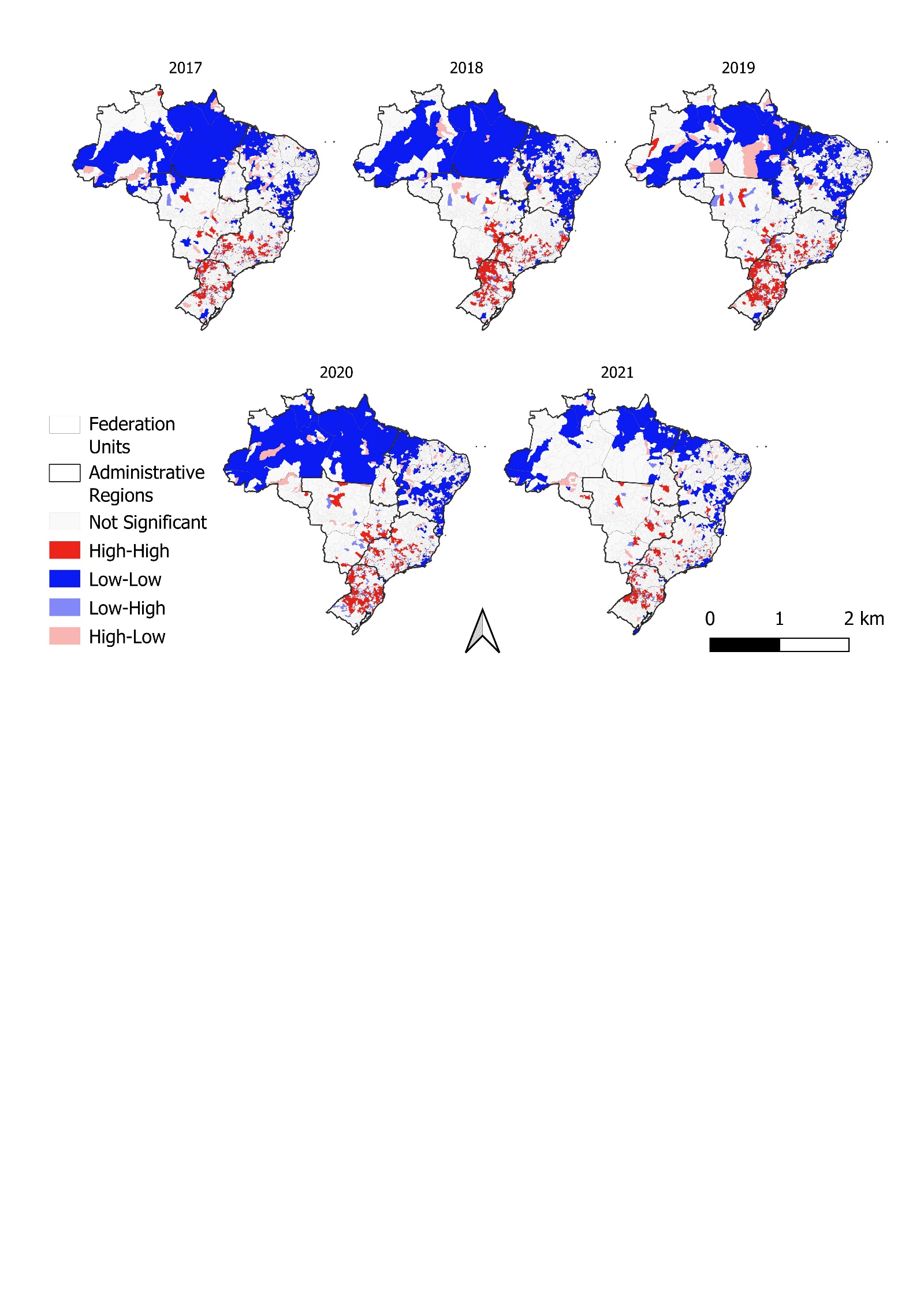

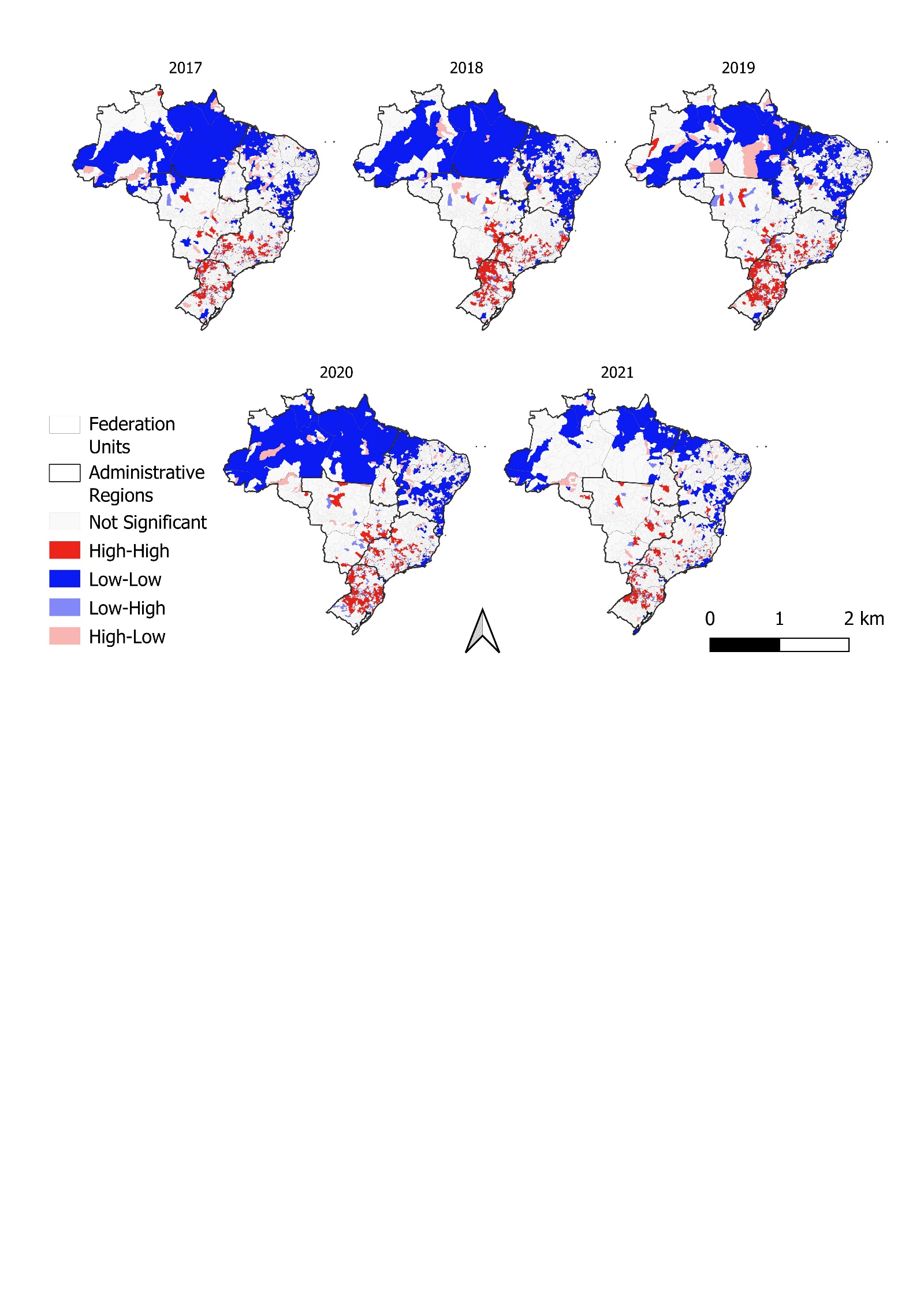

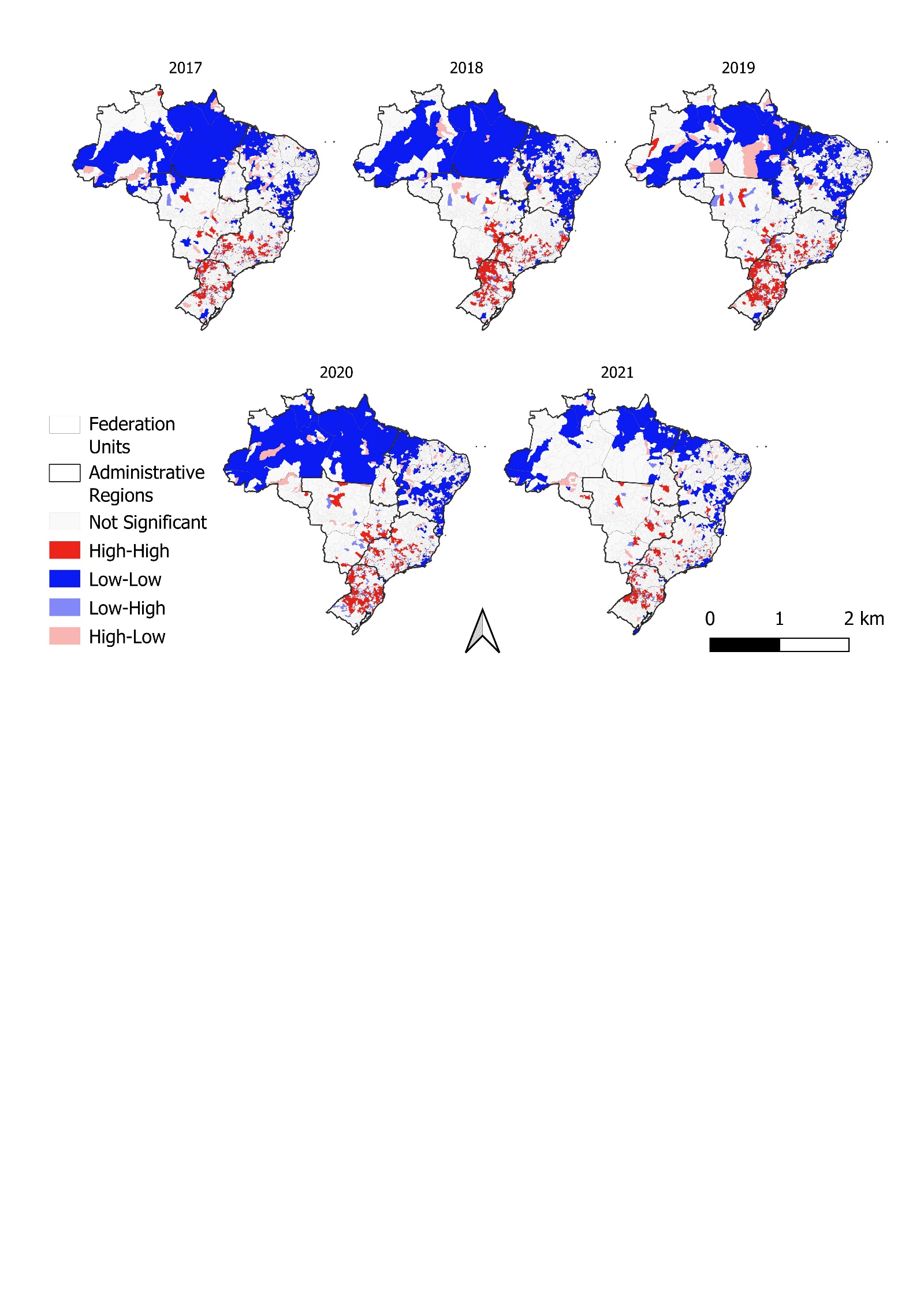


Moran’s I = 0,325

Moran’s I = 0,401

Moran’s I = 0,394

**Not significant (3833)**

**High-High (654)**

**Low-Low (732)**

**Low-High (189)**

**High-Low (156)**

**Not significant (3536)**

**High-High (857)**

**Low-Low (887)**

**Low-High (145)**

**High-Low (139)**

**Not significant (3451)**

**High-High (922)**

**Low-Low (882)**

**Low-High (164)**

**High-Low (145)**

**Not significant (3519)**

**High-High (846)**

**Low-Low (867)**

**Low-High (192)**

**High-Low (140)**

**Not significant (3997)**

**High-High (590)**

**Low-Low (701)**

**Low-High (140)**

**High-Low (136)**


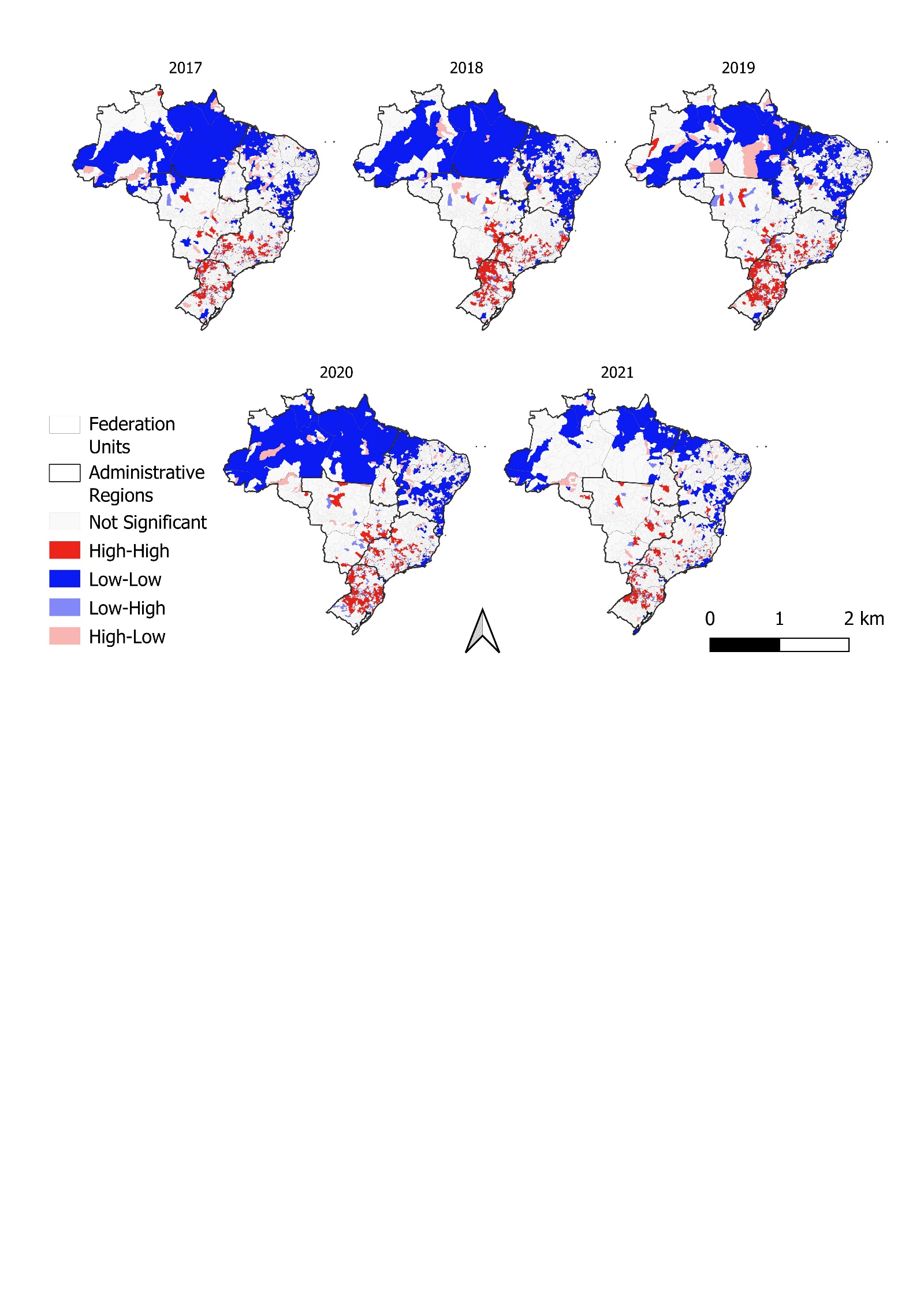

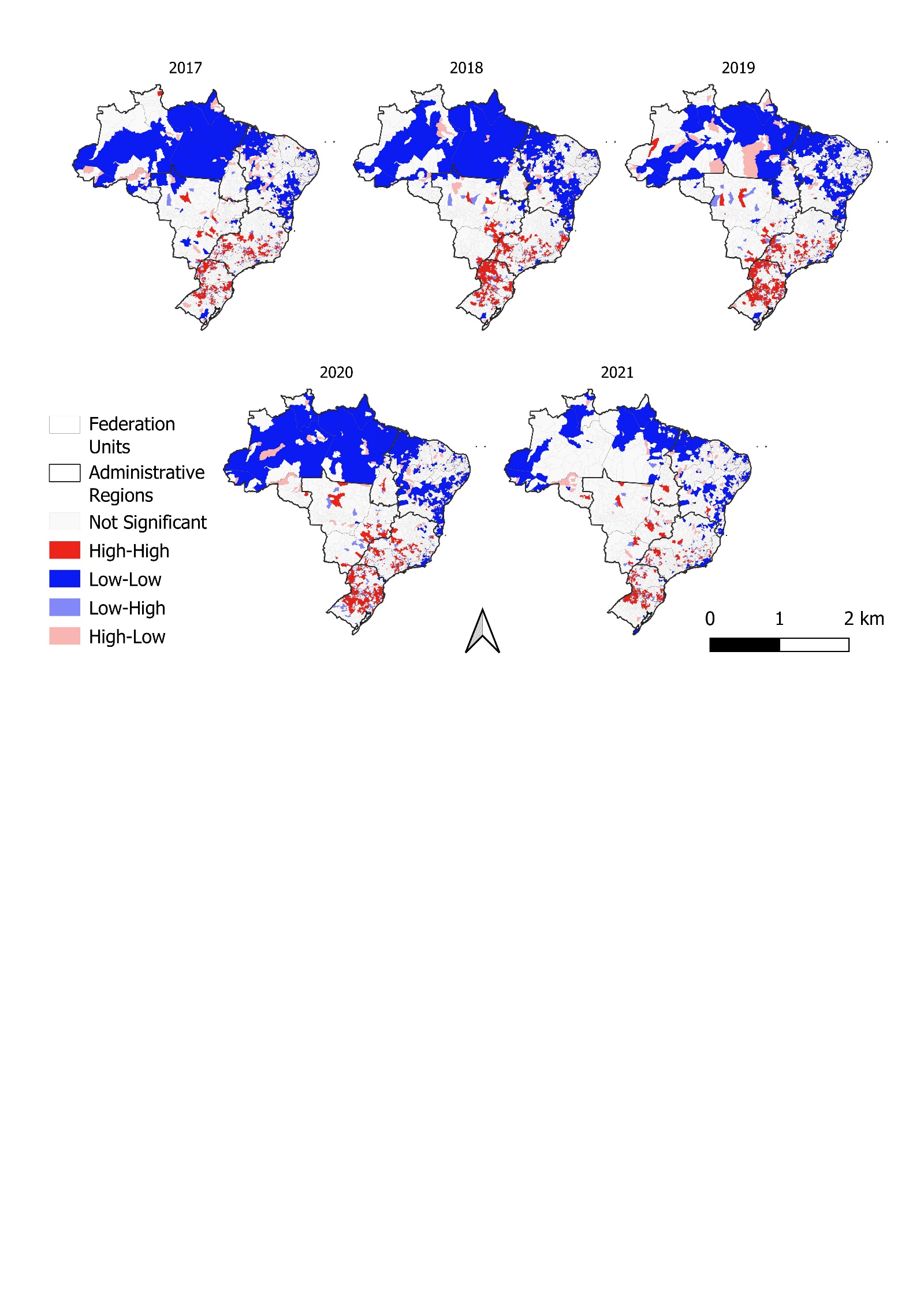


Moran’s I = 0,379

Moran’s I = 0,304


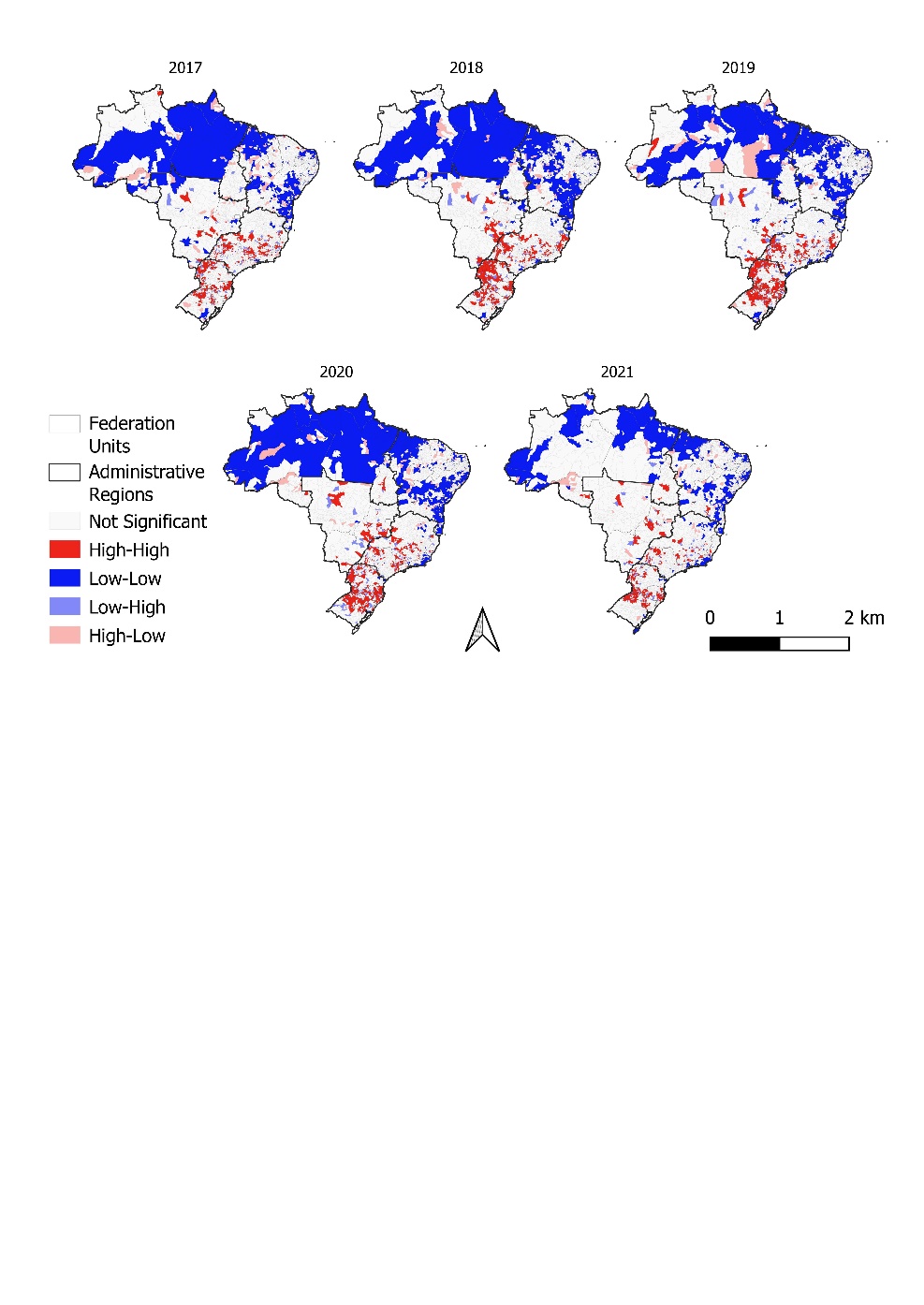


Figure 6S - LISA Cluster Map presenting the distribution of statistically significant spatial clusters of the polio vaccine – Second Booster (fourth year), Brazil, 2017–2021.

Table 1S. Trends of the polio vaccine – First Booster (first year), by Regions and States. National Immunization Program, Brazil, 2011–2021.

| Variable | p-value | Beta | Standard error | Annual percent change | CI* (95%) MIN | CI* (95%) MAX | Tendency |
| --- | --- | --- | --- | --- | --- | --- | --- |
| BRAZIL | <0,001 | -0,020131 | 0,002914 | -4,53 | -6,00 | -3,04 | **Descending** |
| Midwest | <0,001 | -0,021368 | 0,002934 | -4,80 | -6,27 | -3,31 | **Descending** |
| Distrito Federal | 0,023 | -0,0163862 | 0,0056299 | -3,70 | -6,54 | -0,78 | **Descending** |
| Goiás | 0,002 | -0,0208261 | 0,0042760 | -4,68 | -6,82 | -2,49 | **Descending** |
| Mato Grosso | 0,001 | -0,0182158 | 0,0032221 | -4,11 | -5,73 | -2,45 | **Descending** |
| Mato Grosso do Sul | 0,013 | -0,0223876 | 0,0067934 | -5,02 | -8,39 | -1,54 | **Descending** |
| Northeast | <0,001 | -0,0283387 | 0,0042786 | -6,32 | -8,42 | -4,16 | **Descending** |
| Alagoas | 0,054 | -0,0182192 | 0,0078725 | -4,11 | -8,03 | -0,02 | **Stationary** |
| Bahia | <0,001 | -0,0322601 | 0,0049394 | -7,16 | -9,56 | -4,69 | **Descending** |
| Ceará | <0,001 | -0,0239170 | 0,0034405 | -5,36 | -7,07 | -3,61 | **Descending** |
| Maranhão | 0,001 | -0,0335683 | 0,0062691 | -7,44 | -10,47 | -4,31 | **Descending** |
| Paraíba | 0,009 | -0,0261903 | 0,0073175 | -5,85 | -9,44 | -2,12 | **Descending** |
| Pernambuco | <0,001 | -0,0275144 | 0,0040477 | -6,14 | -8,13 | -4,10 | **Descending** |
| Piauí | 0,071 | -0,0127347 | 0,0059890 | -2,89 | -5,93 | 0,25 | **Stationary** |
| Rio Grande do Norte | 0,027 | -0,0212169 | 0,0076428 | -4,77 | -8,56 | -0,82 | **Descending** |
| Sergipe | 0,002 | -0,0281706 | 0,0059912 | -6,28 | -9,22 | -3,25 | **Descending** |
| North | 0,005 | -0,0240564 | 0,0059978 | -5,39 | -8,35 | -2,33 | **Descending** |
| Acre | 0,055 | -0,0208889 | 0,0090971 | -4,70 | -9,19 | 0,02 | **Stationary** |
| Amapá | 0,124 | -0,0429512 | 0,0245402 | -9,42 | -20,48 | 3,19 | **Stationary** |
| Amazonas | 0,005 | -0,0186048 | 0,0046612 | -4,19 | -6,54 | -1,79 | **Descending** |
| Pará | 0,054 | -0,0280897 | 0,0121492 | -6,26 | -12,12 | -0,02 | **Stationary** |
| Rondônia | 0,001 | -0,0185566 | 0,0032828 | -4,18 | -5,84 | -2,50 | **Descending** |
| Roraima | 0,082 | -0,0298643 | 0,0147193 | -6,65 | -13,66 | 0,94 | **Stationary** |
| Tocantins | 0,02 | -0,0063359 | 0,0021134 | -1,45 | -2,55 | -0,34 | **Descending** |
| Southeast | <0,001 | -0,0206217 | 0,0025706 | -4,64 | -5,93 | -3,33 | **Descending** |
| Espírito Santo | 0,001 | -0,0178067 | 0,0034350 | -4,02 | -5,75 | -2,25 | **Descending** |
| Minas Gerais | <0,001 | -0,0122345 | 0,0018721 | -2,78 | -3,74 | -1,81 | **Descending** |
| Rio de Janeiro | 0,001 | -0,0355278 | 0,0062703 | -7,85 | -10,87 | -4,74 | **Descending** |
| São Paulo | <0,001 | -0,0166125 | 0,0025085 | -3,75 | -5,03 | -2,46 | **Descending** |
| South | 0,013 | -0,0096764 | 0,0029361 | -2,20 | -3,72 | -0,67 | **Descending** |
| Paraná | 0,013 | -0,0114676 | 0,0034706 | -2,61 | -4,38 | -0,79 | **Descending** |
| Rio Grande do Sul | 0,273 | -0,0031686 | 0,0026644 | -0,73 | -2,12 | 0,69 | **Stationary** |
| Santa Catarina | 0,002 | -0,0057315 | 0,0012137 | -1,31 | -1,95 | -0,67 | **Descending** |

Note: *CI – Confidence level of Annual Variation (%).
